# Genotyping genome‐edited mutations in plants using CRISPR ribonucleoprotein complexes

**DOI:** 10.1111/pbi.12938

**Published:** 2018-05-29

**Authors:** Zhen Liang, Kunling Chen, Yan Yan, Yi Zhang, Caixia Gao

**Affiliations:** ^1^ State Key Laboratory of Plant Cell and Chromosome Engineering Center for Genome Editing Institute of Genetics and Developmental Biology Chinese Academy of Sciences Beijing China; ^2^ University of Chinese Academy of Sciences Beijing China; ^3^ Key Laboratory of Plant Stress Research College of Life Science Shandong Normal University Jinan China

**Keywords:** PCR/RNP, SpCas9, FnCpf1, TALEN protein, hexaploid wheat, high‐fidelity SpCas9 variants

## Abstract

Despite the great achievements in genome editing, accurately detecting mutations induced by sequence‐specific nucleases is still a challenge in plants, especially in polyploidy plants. An efficient detection method is particularly vital when the mutation frequency is low or when a large population needs to be screened. Here, we applied purified CRISPR ribonucleoprotein complexes to cleave PCR products for genome‐edited mutation detection in hexaploid wheat and diploid rice. We show that this mutation detection method is more sensitive than Sanger sequencing and more applicable than PCR/RE method without the requirement for restriction enzyme site. We also demonstrate that this detection method is especially useful for genome editing in wheat, because target sites are often surrounded by single nucleotide polymorphisms. Using this screening method, we were also able to detect foreign DNA‐free *tagw2* mutations induced by purified TALEN protein. Finally, we show that partial base editing mutations can also be detected using high‐fidelity SpCas9 variants or FnCpf1. The PCR/RNP method is low‐cost and widely applicable for rapid detection of genome‐edited mutation in plants.

## Introduction

Genetic variants are the basic resource for trait improvement and molecular breeding in plants. The use of sequence‐specific nucleases (SSNs), including ZFNs, TALENs, CRISPR/Cas9 and CRISPR/Cpf1 systems, allows one to create double strand breaks at almost any locus and trigger NHEJ or HR repair to generate targeted mutations or insertions (Kim, 2016; Zetsche *et al*., 2017). SSNs have been used in many plant species to create novel genetic resources (Puchta, 2017). Recently, several base editors have been developed that produce targeted C to T or A to G conversions with high efficiency in human cells (Gaudelli *et al*., 2017; Komor *et al*., 2016). The base editing systems have also been shown to generate targeted point mutations in many crops and model plants (Chen *et al*., 2017b; Hua *et al*., 2018; Yan *et al*., 2018; Zong *et al*., 2017).

The outcomes of editing in diploid and polyploid plants are different. When targeting diploid plants, such as rice and maize, four outcomes can be obtained: heterozygous mutants, bi‐allelic mutants, homozygous mutants and mosaic mutants. When targeting polyploid plants, such as wheat, the results are much more complex. Genome editing by both TALENs and the CRISPR/Cas9 system has been successful in wheat (Wang *et al*., 2014). However, its complex allopolyploid nature with three similar but not identical copies of most of its genes makes mutation detection extremely challenging. Recently, we have reported selection‐free editing methods in wheat which need large screening population in T0 generation (Liang *et al*., 2017; Zhang *et al*., 2016). The development of genome editing in wheat calls for an efficient and accurate mutation detection method to identify mutations occurred in the A, B and D genomes.

Current methods for detecting mutations induced by the genome editing toolbox include PCR/RE, T7EI cleavage assay, Sanger sequencing, next generation sequencing (NGS), high‐resolution melting analysis (HRMA) and fluorescent PCR‐capillary gel electrophoresis. Each method has its shortcomings. The PCR/RE method is limited by the requirement that a restriction enzyme site exists in the target site (Shan *et al*., 2014). The CRISPR/Cas9 ribonucleoprotein complex has been developed to overcome this limitation. They used RFLP analysis based on CRISPR/Cas9‐derived RGEN to detect indels induced by SSNs or naturally occurred in cultured cells and animals (Kim *et al*., 2014). The T7EI cleavage assay relies on mismatches in double strand DNA and cannot distinguish homozygous mutant from wild‐type and also fails to distinguish heterozygous mutants with bi‐allelic mutations (Vouillot *et al*., 2015). Its application in hexaploid wheat is also limited by potential SNPs existing near target sites. Sanger sequencing can present direct and detailed information about the mutation types. Bioinformatics online tools, such as DSDecode (Liu *et al*., 2015) and TIDE (Brinkman *et al*., 2014), were developed to decode mutation types from multiple traces of chromatograms derived from PCR amplicons. Genome‐edited mutations can also be identified using NGS followed by software analysis with reliable sensitivity of 0.01%. Bioinformatics tools, such as Cas‐Analyzer (Park *et al*., 2017), CRISPResso (Pinello *et al*., 2016) and CRISPR‐GA (Guell *et al*., 2014), were developed to analyse the NGS sequencing data containing CRISPR/Cas9‐induced mutations in pooling samples. Hi‐TOM is a platform based on NGS for high‐throughput analysis of mutations induced by CRISPR/Cas9 in rice, which can detect mutations from individual plants (Liu *et al*., 2017). However, NGS produces relative short reads and failed to detect large indels. In addition, both Sanger sequencing and NGS are much more expensive when compared with other genotyping method. HRMA (Dahlem *et al*., 2012) and fluorescent PCR‐capillary gel electrophoresis (Ramlee *et al*., 2015) require special instruments.

Here, we employed PCR followed by digestion with purified ribonucleoprotein complexes of SpCas9 or FnCpf1 (hereafter the PCR/RNP method) to detect edited mutations by SSNs in both polyploid and diploid plants. We described the use of this method to detect mutations induced by CRISPR/Cas9 at several target sites in wheat and one target site in rice. We also added a new editing method using purified TALEN protein into the DNA‐free genome editing toolbox of wheat and showed that the PCR/RNP method could be used to detect the resulting mutations. We further demonstrated that various high‐fidelity forms of SpCas9 (including SpCas9‐HF1, HypaCas9, eHF1‐Cas9 and eHypa‐Cas9) could be used to distinguish base edited mutations from wild‐type. Finally, we showed that FnCpf1 could be used in the new method to detect SNPs in the seed region (the first eight PAM‐proximal nucleotide).

## Results

### Establishment of the PCR/RNP mutation detection method

Because the CRISPR nucleases RNP will digest PCR products identical to the sgRNA (wild‐type) but fail to digest PCR products with mutated sequences (mutants). We hypothesized that *in vitro* preassembled CRISPR ribonucleoprotein (RNP) complexes, such as CRISPR/Cas9 and CRISPR/Cpf1, should be able to detect genome‐edited mutations in both polyploid and diploid plants. As a first step, we tested if PCR amplicons containing wild‐type target sites were completely cleaved by the corresponding preassembled RNP complexes. SpCas9, AsCpf1 and FnCpf1 proteins were expressed in *Escherichia coli* and purified with C‐terminal 6*His tag, respectively (Figure S1). In the RNP‐mediated *in vitro* cleavage method, only the guide‐RNA (sgRNA for SpCas9 and crRNA for FnCpf1 and AsCpf1) needs to be replaced when the target site is changed. To identify the best conditions for the PCR/RNP method using SpCas9, we selected six sgRNAs (sg‐TaGW2 for *TaGW2*, sg‐TaCer9 for *TaCer9* and sg‐OsPDS1~4 for *OsPDS*) targeting three genes of wheat and rice (Table S1) and examined the effects of incubation time and RNP dosage on the cleavage of wild‐type PCR amplicons. We found that increasing the incubation time had little effect on digestion (Figure S2a) and the minimum RNP dosage required for total digestion depended on the sgRNA activity (Figure S2b). A dosage of RNP (500 ng) and a long incubation time (2–3 h) were used in further experiments and reproducibly achieved complete digestion of wild‐type PCR amplicons for many targets. Similar results were obtained with AsCpf1 and FnCpf1 (Figure S2c).

SSNs always produce small deletions or insertions at the target site if the DSBs are repaired via the NHEJ pathway (Gaj *et al*., 2013). We tested whether the PCR/RNP method differentially cleaved PCR amplicons containing wild‐type and target sites edited by these SSNs. The OsPDS‐1 target site was selected, as it contains PAM sequence for Cpf1 (5′‐TTTG‐3′ for AsCpf1 and 5′‐TTG‐3′ for FnCpf1) at its 5′‐end and for SpCas9 (5′‐AGG‐3′) at its 3′‐end (Figure S3a). Mutations occurred regions induced by Cpf1 (13–23 nucleotides distal to the PAM site) (Zetsche *et al*., 2015) and by Cas9 (three nucleotides proximal to the PAM site) (Cong *et al*., 2013; Mali *et al*., 2013) were consistent in the OsPDS‐1 target site. Therefore, a series of PCR products containing 1–6 bp deletions in the mutation occurred regions were created to test the PCR/RNP method. None of the PCR amplicons used, containing 1–6 bp deletions, was cut by the SpCas9 and FnCpf1 RNP complexes (Figure S3b), and the AsCpf1 RNP complexes had weak cleavage activity against the alleles with 1–3 base deletions (Figure S3b). On the other hand, all the wild‐type PCR amplicons were cut. The majority mutations induced by Cpf1 are deletions and ranged from 6 to 13 bases in size (Tang *et al*., 2017). Therefore, the weak cleavage activity of AsCpf1 against alleles with 1–3 base deletions will not impact the PCR/RNP methods for analysis of mutations induced by Cpf1. Above results showed that both Cas9 and Cpf1 RNPs can be used to detect indels.

**Figure 1:**
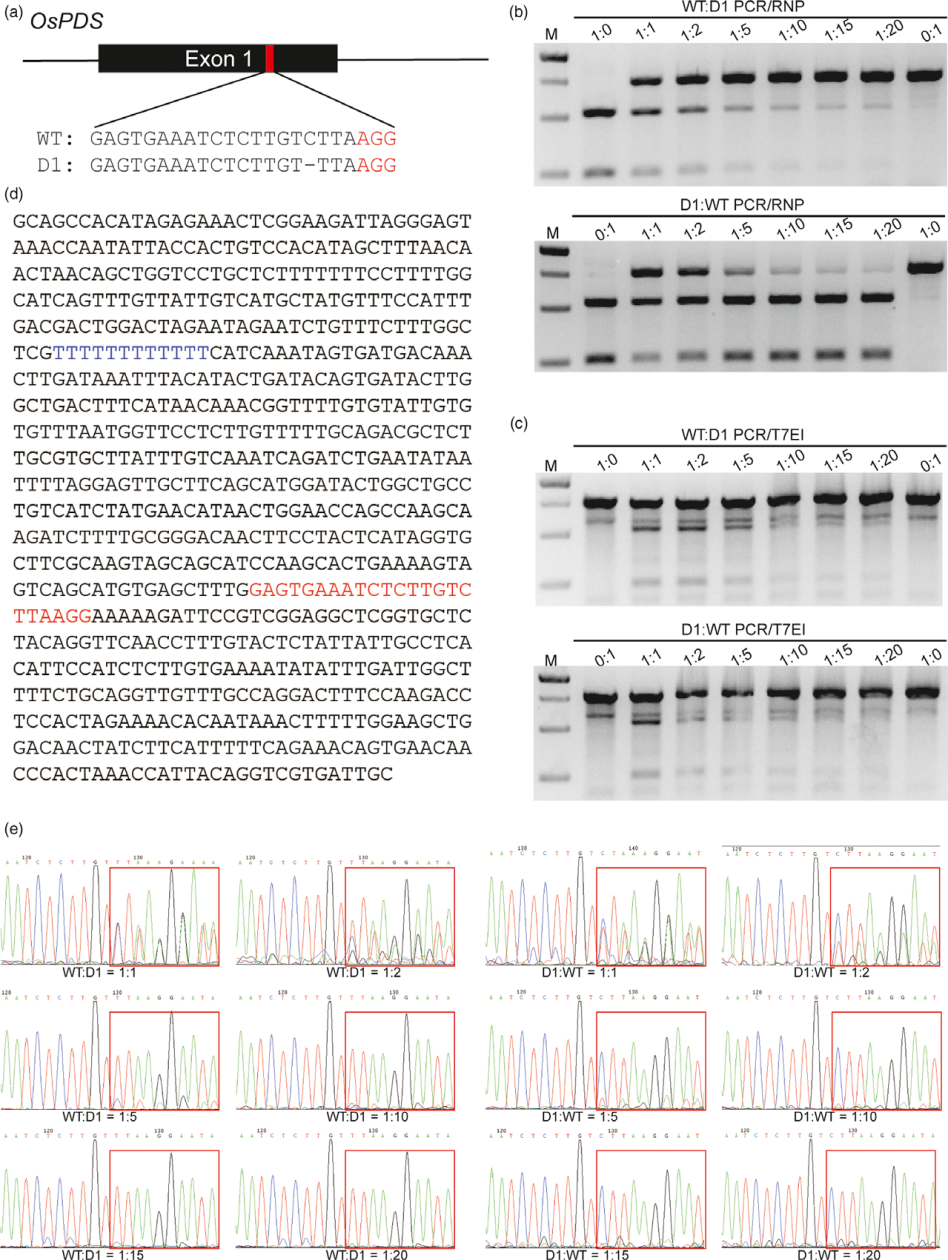
Comparison of the sensitivities of mutation detection by PCR/RNP, PCR/T7EI and direct Sanger sequencing. (a) The sg‐OsPDS‐1 target site is located in exon1 of *OsPDS*. The PAM sequence is highlighted in red. ‘D1’ indicates a 1 bp deletion at the target site. (b & c) Mixtures of WT and D1 PCR amplicons in different ratios were treated by PCR/RNP and PCR/T7EI. (d) DNA sequence of the PCR amplicons surrounding the sg‐OsPDS‐1 target site. The sgRNA sequence and 12 consecutive T's in the amplicons are highlighted in red and blue, respectively. (e) Mixtures of different proportions of WT and D1 PCR amplicons sequenced by the Sanger method.

Sensitivity is one of the most important criteria for mutation detection systems. To quantify the sensitivity of the PCR/RNP method, a series of mixtures with different ratios of wild‐type (WT) and 1‐bp deletion (D1) PCR amplicons in the sg‐OsPDS‐1 target site (Figure 1a) were subjected to CRISPR/Cas9 RNP cleavage. The mixtures were also subjected in parallel to T7EI cleavage and Sanger sequencing. The PCR/RNP method detected the deletion efficiently in mixtures with ratios ranging from 1:1 to 1:20 of WT:D1 and of D1:WT (Figure 1b). While T7EI cleavage assay failed to distinguish samples with the same ratios of WT:D1 and D1:WT, because they will result in the same ratio of heteroduplex DNA after annealing (Figure 1c). This is an evidence that T7EI assay may underestimate mutagenesis frequencies, which is previously reported (Sentmanat *et al*., 2018). We also found that samples containing only WT or D1 PCR amplicons were partially cut by T7EI (Figure 1c). This was probably due to the 12 consecutive T's near the target region, which may result in base deletions during PCR amplification (Figure 1d). Sequencing is the most informative method of mutation detection. However, direct Sanger sequencing failed to detect mutations in mixtures of WT:D1 and D1:WT of 1:10, 1:15 and 1:20 (Figure 1e). These findings demonstrate that the PCR/RNP method is superior to T7EI assay in terms of accuracy, and superior to Sanger sequencing in terms of sensitivity.

### Using the PCR/RNP method to screen mutants in plants

**Figure 2:**
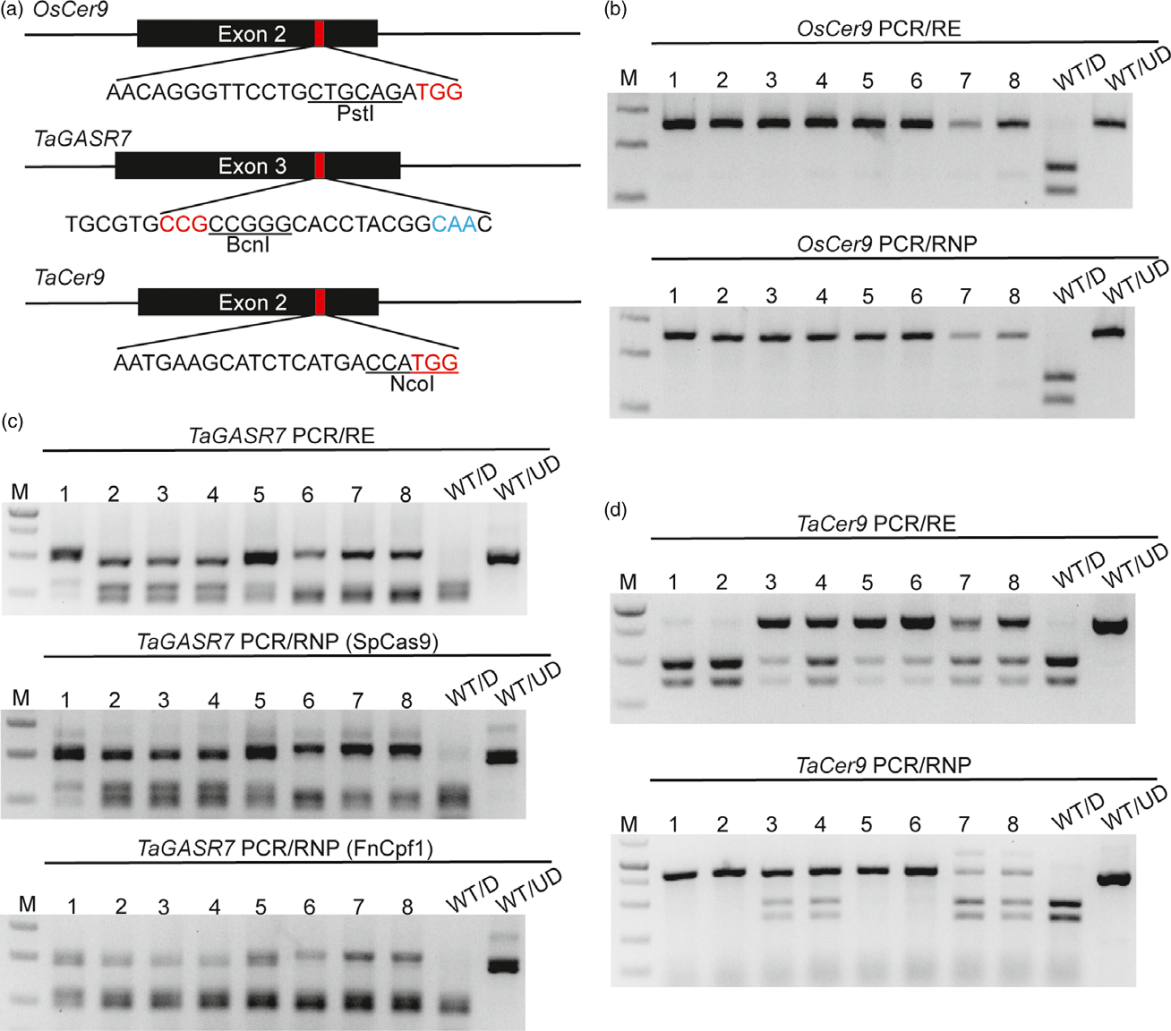
Genotyping of mutants induced by CRISPR/Cas9 reagents using PCR/RNP and PCR/RE in plants. (a) Three sgRNAs were designed to target rice *OsCer9* and wheat *TaGASR7* and *TaCer9*, respectively. The PAM sequences are highlighted in red and the corresponding restriction enzyme sites are underlined and labelled. The PAM sequence of FnCpf1 (5′‐CAA‐3′) in the sg‐TaGASR7 is highlighted in blue. (b) The results of PCR/RE and PCR/RNP assays to detect eight homozygous *oscer9* mutants induced by CRISPR/Cas9. (c) The results of PCR/RE and PCR/RNP (SpCas9 and FnCpf1) assays to detect eight representative mutants induced by gasr7‐IVTs. (d) Agarose gel analysis of eight CRISPR/Cas9‐induced *tacer9* mutants detected by PCR/RE and PCR/RNP. ‘WT/D’: wild‐type amplicons digested with T7EI, restriction enzyme or CRISPR/Cas9 RNP; ‘WT/UD’: wild‐type amplicon not digested.

The final application of the PCR/RNP method examined was aimed at rapid detection of mutations at the plant level independent of the presence of a restriction enzyme site. We thus used PCR/RNP to detect mutations induced by CRISPR/Cas9 reagents in rice (*OsCer9*) and hexaploid wheat (*TaGASR7*,* TaGW2* and *TaCer9*). All the four sgRNA targets tested contained available restriction enzyme sites. For targets of *OsCer9*,* TaGASR7* and *TaGW2*, the restriction enzyme sites perfectly overlap with the Cas9 cutting site; for *TaCer9*, the restriction enzyme site is tightly adjacent to the cutting site (Figures 2a and S4a). Eight representative mutants in each target region were analysed in parallel by PCR/RE and PCR/RNP. In the case of *TaGASR7*, the mutants were also analysed using equivalent FnCpf1 RNPs. The genotypes of *OsCer9*,* TaGASR7* and *TaGW2* detected by the PCR/RE and PCR/RNP methods were the same (Figures 2b,c and S4). Gel analysis of *tagw2* mutants with PCR/RNP yielded one more band than PCR/RE in all the tested plants including the wild‐type. This may have been due to gel‐shift of the DNA‐RNP complexes that were formed. However, the additional band did not affect identification of the genotypes (Figure S4).

**Figure 3:**
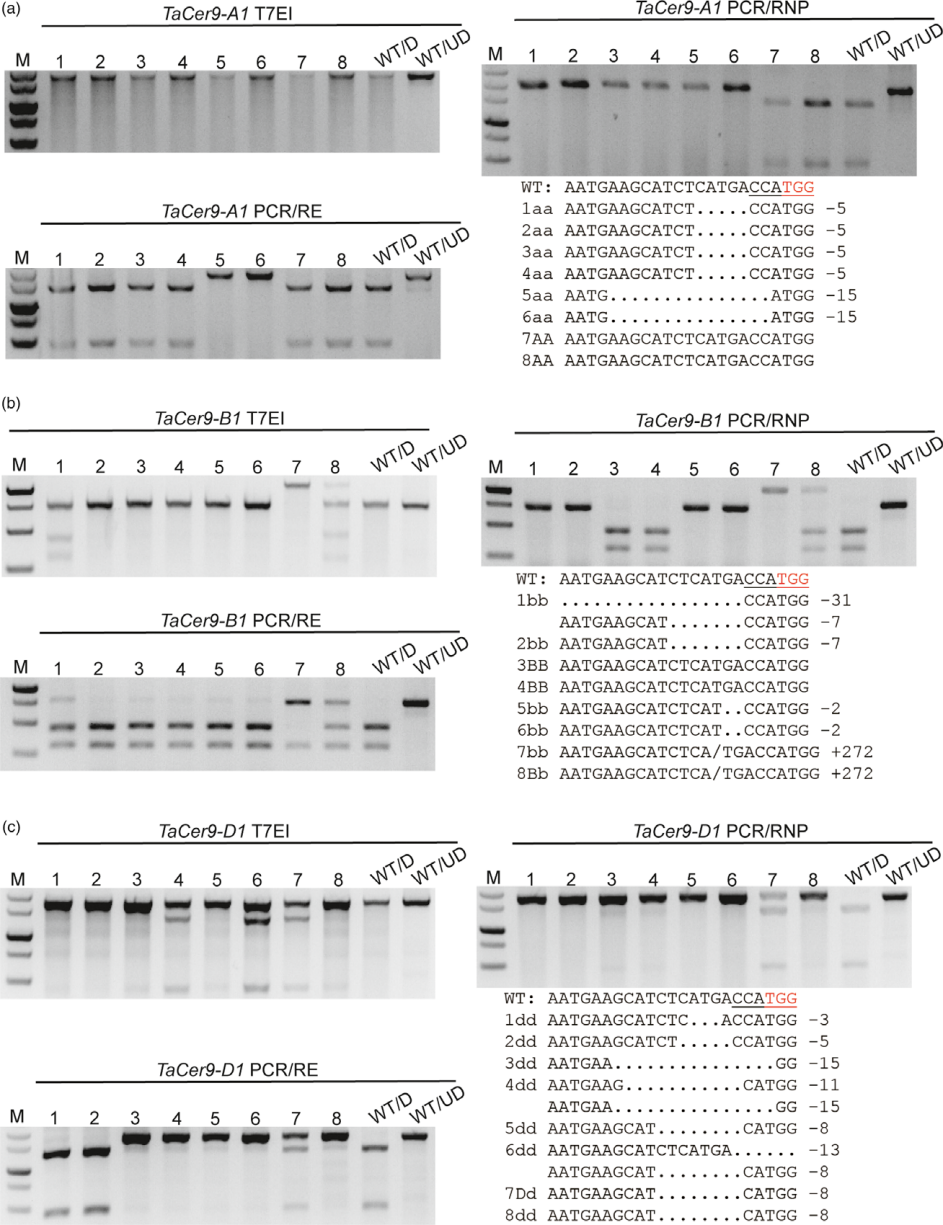
Mutant identification in *TaCer9* in hexaploid wheat using homoeologue‐specific primer sets. Agarose gel showing eight representative *tacer9* mutants in *TaCer9‐A1* (a), *TaCer9‐B1* (b) and *TaCer9‐D1* (c) detected by the three methods. Genotypes and mutant patterns were confirmed by Sanger sequencing. Hyphens denote deleted nucleotides. The PAM motif (5′‐TGG‐3′) is shown in red. The NcoI restriction enzyme site (5′‐CCATGG‐3′) is underlined.

One exception is the sg‐TaCer9 target site. The genotyping results obtained by PCR/RE and PCR/RNP were different. We supposed that this may be due to the fact that many CRISPR/Cas9‐induced indels, such as deletions of DNA bases located at the 5′ end of the preferred CRISPR/Cas9 cut site or insertions, will not be detectable by PCR/RE. To test the hypothesis, we analysed eight *tacer9* mutants using homoeologue‐specific primer sets (Table S2). As expected, T7EI assay failed to distinguish homozygous mutations from wild‐type and the PCR/RE assay missed mutations associated with intact restriction enzyme sites. Only the PCR/RNP method yielded the similar results as Sanger sequencing (Figure 3). Mutation types induced by tacer9‐RNPs were further verified in wheat protoplasts. We found that the mutation frequency measured by PCR/RNP (28.9%) was higher than that by PCR/RE (11.3%) (Figure S5a). We gel purified both undigested bands from the PCR/RNP and PCR/RE assay and sequenced them by cloning. The sequencing results showed that the majority of mutations (21/24) detected by PCR/RNP retained the NcoI site (Figure S5b), whereas most of the mutations (6/7) detected by PCR/RE had lost the NcoI site (Figure S5c), which indicates that the PCR/RE method fails to detect mutations that do not remove the restriction enzyme site. We also analysed the mutations in protoplasts by T7EI assay. However, all the samples gave rise to smeared DNA bands whether or not they were exposed to tacer9‐RNPs (Figure S5a). This was because a conserved primer set recognizing all three homoeologues (*TaCer9‐A1*,* ‐B1* and *‐D1*) simultaneously was used, and there are several SNPs in the amplified region (Figure S6). This example indicated that when using the PCR/RE method, the restriction enzyme sites should be perfectly cover the Cas9 cutting site. Otherwise, some small indels will be overlooked. This feature further limited choices of candidate sgRNA target sites. The PCR/RNP method thus has clear cut advantages over the T7EI and PCR/RE methods, especially for polyploid plants such as wheat.

### PCR/RNP analysis of mutations induced by purified TALEN protein

**Figure 4:**
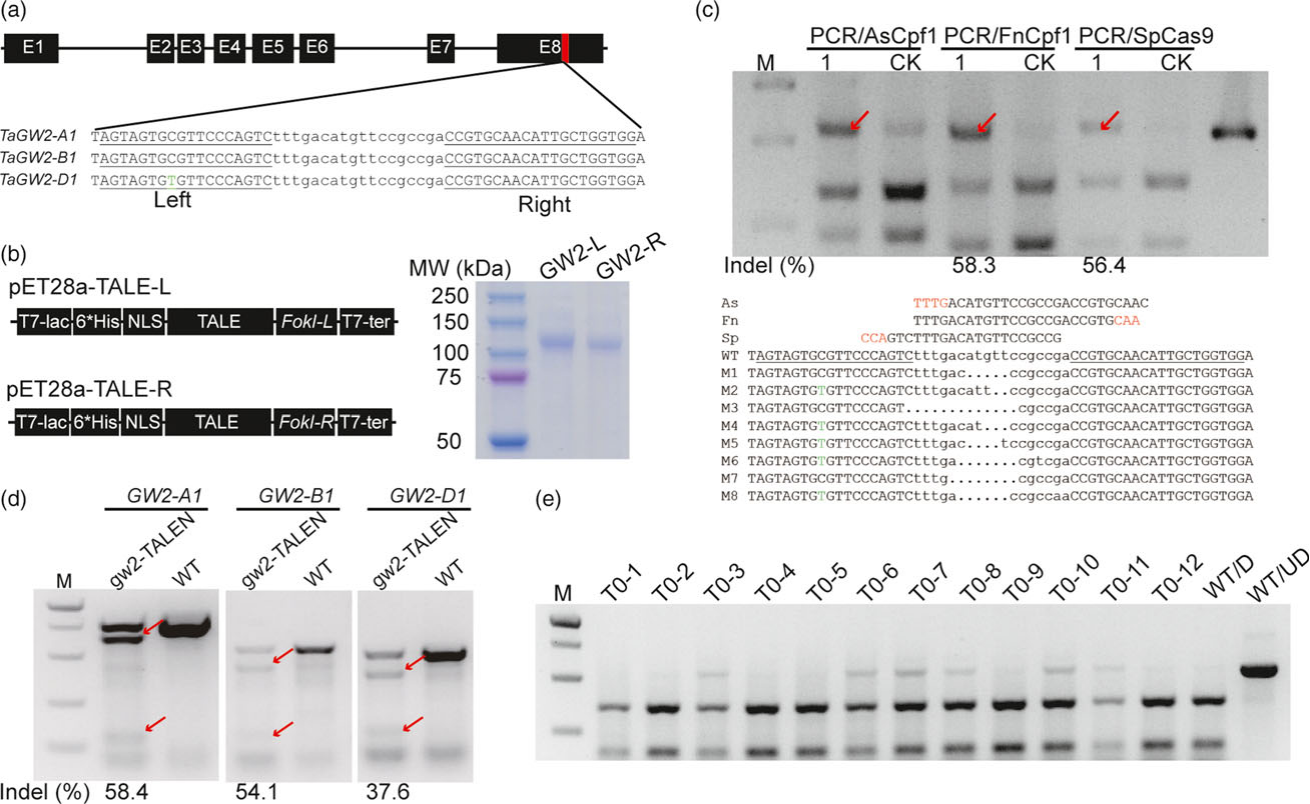
Targeted mutagenesis at the *TaGW2* locus using purified TALEN protein. (a) The gene architecture of *TaGW2* and the TALEN target site in exon 8. The left and right target sequences are underlined and the spacer sequence is in lower case. The SNP in the target sequence of *TaGW2‐D1* is highlighted in green. (b) Diagram of the plasmid used for bacterial expression and purification (left). SDS‐PAGE of the purified GW2‐L and GW2‐R proteins (right). (c) Mutation frequency at the gw2‐TALENs target site in protoplasts detected by the PCR/RNP method using different RNA‐guided endonucleases with a conserved primer set. ‘1’ indicates protoplasts incubated with the gw2‐TALENs protein, and ‘CK’ is a negative control. (d) Mutation frequency induced by purified gw2‐TALENs protein in protoplasts detected by the PCR/T7EI method with homoeologue‐specific primer sets. The red arrows indicate mutant bands. (e) Outcome PCR/RNP to detect TALEN‐induced mutations in 12 representative T0 plants. ‘WT/D’: wild‐type amplicons digested with CRISPR/Cas9 RNP, ‘WT/UD’: wild‐type amplicon not digested.

A pair of TALEN was designed to target the three homoeologues of *TaGW2* simultaneously (Table S3). The gw2‐TALENs target a conserved region in exon 8 and match perfectly their recognition sites in *TaGW2‐A1* and *‐B1*, whereas there is a single nucleotide mismatch in the left binding site of *TaGW2‐D1* (Figure 4a). The two TALEN monomers (GW2‐L & GW2‐R) were cloned into the bacterial expression plasmid (pET‐28a), expressed in *E. coli* and purified with N‐terminal 6*His tag, respectively (Figures 4b and S7a). In the *in vitro* cleavage assay, they had robust activity against PCR amplicons containing the TALEN recognition sites (Figure S7b). The purified gw2‐TALEN proteins were then delivered into wheat protoplasts by PEG‐mediated transfection. We designed three different RNPs incorporating AsCpf1, FnCpf1 and SpCas9, respectively, to detect the mutations by the PCR/RNP method (Table S1). The three target sites were located in the spacer region of the TALEN target sequence. Our results showed that FnCpf1 and SpCas9 detected the mutations while the wild‐type sequence was cut (Figure 4c); on the other hand, the AsCpf1 RNPs failed to completely cut the wild‐type sequence. The mutation frequencies measured by FnCpf1 and SpCs9 RNPs (58.3% and 56.4%) were similar. The undigested bands were further gel purified and confirmed by Sanger sequencing of single colonies (Figure 4c). The mutation frequencies determined using T7EI at *TaGW2‐A1*,* TaGW2‐B1* and *TaGW2‐D1* were comparable (58.4%, 54.1% and 37.6%) and consistent with the results by the PCR/RNP method (Figure 4d).

Having confirmed that the purified gw2‐TALEN proteins were highly active in wheat protoplasts, we next delivered them into immature wheat embryos via particle bombardment. As we previously described for transient expression of CRISPR/Cas9 reagents, no herbicide selection was used in the entire tissue culture procedure (Zhang *et al*., 2016). We used the PCR/RNP method to screen mutations induced by the gw2‐TALENs in the T0 generation, using CRISPR/Cas9 RNPs against the spacer region of the TALEN target site (Figure 4d and Table S1). We used PCR/RNP together with previously described pooling method for mutation screening (Zhang *et al*., 2016). In total, six mutants were detected from 338 pools of regenerated seedlings (Figure 4e). The six mutants were further analysed with homoeologue‐specific primer sets (Table S2). All the mutations were located in the *TaGW2‐A1* locus (Figure S8).

### PCR/RNP analysis of mutations generated by base editors

**Figure 5:**
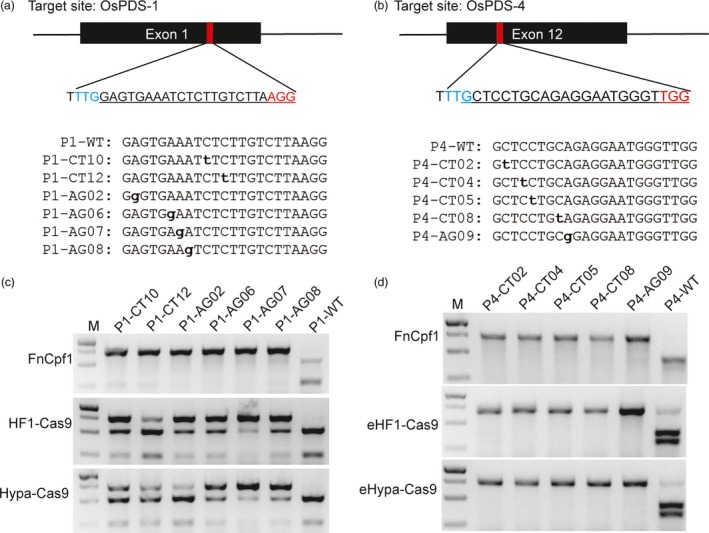
Genotyping base editing mutations by the PCR/RNP method. (a & b) The OsPDS‐1 and OsPDS‐4 target sites in exon1 and exon12 of *OsPDS*, respectively. The PAM sequences of FnCpf1 and SpCas9 are highlighted in blue and red, respectively. Candidate C to T and A to G conversions in the base editing windows are listed. (c & d) Analysis of base editing mutations in both target sites induced by FnCpf1 and the high‐fidelity SpCas9 variants.

Base editing systems enable highly efficient conversion of C to T or A to G in a programmable manner and have created a new era of genome editing, especially for gain‐of‐function analysis (Kuscu and Adli, 2016). To investigate whether the PCR/RNP method can be used to genotype single SNPs, we constructed two series of SNPs located in the target sites of OsPDS‐1 and OsPDS‐4 (Figure S9a and b). Both target sites have 5′‐terminal PAM sequence for Cpf1 (AsCpf1 and FnCpf1) and 3′‐terminal PAM sequences for SpCas9. PCR amplicons containing these SNPs were treated with RNP complexes of SpCas9, AsCpf1 and FnCpf1 containing wild‐type sgRNA and crRNA, respectively. Unlike the situation for genome editing *in vivo*, SNPs located in all positions of the target sequence (except the GG sequence for the PAM) – even in the seed region – were totally digested by SpCas9 RNPs, whereas AsCpf1 and FnCpf1 RNPs were sensitive to SNPs located in the first eight PAM‐proximal nucleotides (M1‐M8 for OsPDS‐1 and M2‐M9 for OsPDS‐4). Several SNPs located in other regions (M10, M12 M15 and M17) of OsPDS‐1 also failed to be digested by FnCpf1 RNPs is just case‐by‐case (Figure S9c and d). Therefore, we propose that SNPs located in the target seed regions of Cpf1 can be efficiently detected by the PCR/RNP method. This inference was confirmed by testing candidate base editing changes (C to T and A to G) in the targeting windows of OsPDS‐1 and OsPDS‐4 target sites using the corresponding FnCpf1 RNP complexes (Figure 5).

Recently, a number of high‐fidelity SpCas9 variants, including eSpCas9 1.0, eSpCas9 1.1, SpCas9‐HF1 and HypaCas9, have been rationally engineered to increase the specificity of editing (Chen *et al*., 2017a; Kleinstiver *et al*., 2016; Slaymaker *et al*., 2016). By combining the amino acid changes in eSpCas9 1.1 with SpCas9‐HF1 and HypaCas9, we created new enhanced HF1‐Cas9 (eHF1‐Cas9) and enhanced hyper‐accurate Cas9 (eHypa‐Cas9) variants. We expressed and purified these high‐fidelity SpCas9 variants to detect SNPs using the PCR/RNP method (Figure S1). The six high‐fidelity SpCas9 forms together with wild‐type sgRNA were used to cleave PCR amplicons containing mismatches at each position of the OsPDS‐1 and OsPDS‐4 target sites (Figure S9a and b). SpCas9‐HF1 and HypaCas9 had almost no cleavage activity for SNPs in the distal regions of the OsPDS‐1 PAM (M1–M8), but completely cut the wild‐type (Figure S10). However, they had robust cleavage activity for all the SNPs in the OsPDS‐4 target site (Figure S11). We further found that eHF1‐Cas9 and eHypaCas9 had no cleavage activity for SNPs in the distal region of the OsPDS‐4 PAM (M2–M9), but robust cleavage activity for the wild‐type (Figure S11). The base editing windows of C to T and A to G mainly ranged from positions 4–8 in the protospacer (Gaudelli *et al*., 2017; Komor *et al*., 2016). Therefore, we supposed that the high‐fidelity SpCas9 variants could be used to detect mutations generated by base editing, and we tested candidate base editing changes (C to T and A to G) at both target sites (OsPDS‐1 and OsPDS‐4) (Figure 5c and d). The results showed that PCR/RNP is less effective in detecting SNPs than in detecting indels, but the latter can at least be distinguished from the wild‐type sequence.

## Discussion

An effective genotyping method is very important for genome editing in plants. Previously, we mainly used the PCR/RE method for mutant screening (Shan *et al*., 2014). However, the available restriction enzyme sites limited the choice of target sites. Here, we have described a PCR/RNP method based on the RNA‐guided endonucleases SpCas9 or FnCpf1 for identifying indels generated by CRISPR/Cas9 that overcomes this limitation of the PCR/RE method. Unlike the use of T7EI, the PCR/RNP method can distinguish homozygous mutants from wild‐type and also bi‐allelic from heterozygous mutations. Another advantage of PCR/RNP over the T7EI method is that mutation detection with RNPs is not affected by background noise (SNPs) around the target site, which has great significance for polyploid plants such as wheat. Sanger sequencing is the most informative method for mutation detection in plants. However, this is much costlier, especially for low mutation frequencies or large populations of testable seedlings. Direct sequencing to detect mutations in hexaploid wheat needs homoeologue‐specific PCR, and specific primers are not always easy to design. Furthermore, Sanger sequencing is less sensitive than PCR/RNP (Figure 1b and e) and so may overlook some mutations when a pooled screening approach is used.

Genome editing approaches without the involvement of any foreign DNA is one of the directions for optimization. TALEN or CRISPR/Cas9 delivered as RNA or protein can function with a transiently manner without integrated into the host genome. In this study, we report the successful delivery of purified TALEN proteins into immature wheat embryos and targeted mutagenesis at the *TaGW2* locus in the T0 generation. Mutagenesis induced by TALEN protein can also be detected using the PCR/RNP method (Figure 4). We showed that the PCR/RNP method is especially suitable for the selection‐free genome editing procedures, including TECCDNA, TECCRNA (Zhang *et al*., 2016) and TECCRNP (Liang *et al*., 2017) procedures that we previously developed in wheat. Because no selective antibiotic is used in the tissue culture process, large numbers of seedlings may need to be tested in the T0 generation. We also propose that indels whether naturally occurred or induced by other sequence‐specific nucleases could be easily identifiable by the PCR/RNP method, the only requirement being the presence of GG or TT (CC or AA on the complementary strand) in the PAM sequences for SpCas9 and FnCpf1.

An important factor in evaluating a mutation detection method is how widespread is its application. For ease of operating the PCR/RNP method, we used crude PCR products without purifying and quantifying them, and an excess of RNP (500 ng for each reaction). Sufficient *in vitro* transcribed and purified sgRNA or crRNA can be easily obtained from one transcription reaction using a commercial kit to support over 300 cleavage reactions. High yields of purified active SpCas9 and FnCpf1 proteins can be obtained in 3–4 days. Usually, over 4 mg of SpCas9 or FnCpf1 can be obtained from 1L of induced bacterial cells, and the proteins can be stored at −80°C for several months. The entire mutation detection procedure using PCR/RNPs can be completed in a few hours and the results can be displayed by standard agarose gel analysis.

Single nucleotide polymorphisms are very important genetics resources for generating elite traits in crop plants (Rafalski, 2002). Unlike for indel detection, there is a difference between single nucleotide mismatch cleavage assays based on SpCas9 versus FnCpf1: a mismatch at any position of the protospacer does not prevent cutting by SpCas9, but it does prevent cutting by FnCpf1 (Figure S9). This characteristic makes SpCas9 suitable for screening mutations at target sites with one‐mismatch at the same time as at target sites with no mismatch. For example, sg‐TaGW2, which we previously used to target *TaGW2*, perfectly matched *TaGW2‐B1* and *‐D1*, but had a single nucleotide mismatch at *TaGW2‐A1* (Zhang *et al*., 2016), and mutations occurring in all three homoeologues could be simultaneously detected by the PCR/RNP using a conserved primer set (Figure S4). In the case of FnCpf1, a single nucleotide mismatch in the seed region (the first eight PAM‐proximal nucleotides) prevented cutting by RNP containing the wild‐type crRNA. This may be one explanation why Cpf1 has higher specificity than SpCas9 (Kim *et al*., 2016). Therefore, SNPs, whether naturally occurred or generated by base editing, located in the seed regions of target sites for FnCpf1 can be effectively detected by the PCR/RNP method.

In summary, we describe an efficient mutant screening method based on RNP of CRISPR/Cas9 or CRISPR/Cpf1 system in plants. The PCR/RNP method is fast, cheap and easy to apply without the need for appropriate restriction enzyme sites and special instruments. In addition, we describe for the first time an alternative DNA‐free genome editing procedure in wheat using purified TALEN proteins and show that the resulting mutations can also be identified by the PCR/RNP method. Our works provided powerful tools not only for high‐throughput detection requirements in plants but also for accurately identify the mutation in different genome of polypoid plants even in complicated genetic background.

## Experimental procedures

### Production of sgRNA and crRNA

Templates for transcription of sgRNA and crRNA were amplified using corresponding primer sets (Table S2) with high‐fidelity DNA polymerase and purified with PCR Clean‐Up Kit (Axygen). sgRNA and crRNA were synthesized using the HiScribe T7 *In Vitro* Transcription Kit (New England Biolabs) according to the manufacturer's instructions and purified by ethanol precipitation method. The concentration was analysed using NanoDrop spectrophotometer.

### Purification of high‐fidelity SpCas9 variants and Cpf1 orthologous

Point mutations were introduced into the Cas9 coding sequences of pET28a‐Cas9‐His to construct bacterial expression plasmids carrying eSpCas9 1.0 (K810A, K1003A, R1060A), eSpCas9 1.1 (K848A, K1003A, R1060A), SpCas9‐HF1 (N497A, R661A, Q695A, Q926A), HypaCas9 (N692A, M694A, Q695A, H698A), eHF1‐Cas9(N497A, R661A, Q695A, K848A, Q926A K1003A, R1060A) and eHypaCas9 (N692A, M694A, Q695A, H698A, K848A, K1003A, R1060A) (Figure S1a). These high‐fidelity SpCas9 variants were expressed in *E. coli Rosetta* and purified as previously described for Cas9 (Liang *et al*., 2017). The AsCpf1 and FnCpf1 bacterial expression plasmids were kindly provided by Dr. Jin‐Soo Kim. The purification procedure for AsCpf1 and FnCpf1 proteins was the same as for Cas9 protein, while dialyzed with Cpf1 storage buffer (20 mM HEPES pH 7.5, 200 mM NaCl and 1 mM DTT). The purities and concentrations of the purified proteins were measured by SDS‐PAGE (Figure S1b) and the Bradford protein assay, respectively.

### Creation of PCR products for *in vitro* cleavage assay

Wild‐type PCR products containing OsPDS‐1 and OsPDS‐4 were amplified using high‐fidelity DNA polymerases and cloned into pEasy‐Blunt (TransGen Biotech, Beijing) vector. 1~6 bp deletions for OsPDS‐1 (D1~D6) and series SNPs for OsPDS‐1 and OsPDS‐4 target sites (Figure S9a and b) were produced by Fast Mutagenesis System (TransGen Biotech, Beijing). PCR products were amplified by 32–35 cycles with Taq DNA polymerases.

### PCR/RNP method

The PCR products (2–3 μL depending on the concentration) were digested with the corresponding RNP complexes in Cas9 reaction buffer (20 mM HEPES, pH 7.5, 150 mM KCl, 10 mM MgCl_2_, 0.5 mM DTT) in a total volume of 10 μL. For each reaction, 2–3 μL PCR products, 0.5 μg Cas9 or Cpf1 protein and 0.5 μg sgRNA or crRNA were mixed and ddH_2_0 were added up to 10 μL. The mixtures were firstly incubated at 37°C for 3 h for cleavage and then incubated at 65°C for 10 min to stop the reaction, and the products were analysed immediately on 2% agarose gel.

### Purification of TALEN protein

Fragments containing the SV40 NLS and FokI‐L or FokI‐R were cloned into pEasy‐Blunt Simple to yield plasmids pLZ‐NLS‐FokI‐L and pLZ‐NLS‐FokI‐R. Golden Gate assembled TALEN Left/Right was released by digestion with XbaI and BamHI and inserted into pLZ‐NLS‐FokI‐L (XbaI/BamHI) and pLZ‐NLS‐FokI‐R (NheI/BamHI), respectively. Then, the intact TALEN monomers were cloned into bacterial expression plasmid pET‐28a by digestion with SacI/XhoI. The monomer TALEN proteins were expressed in *E. coli Rosetta* at 22°C, purified by nickel affinity chromatography (GE Healthcare, cat. no. 17‐5318‐01) and dialyzed with TALEN storage buffer (20 mM HEPES pH 7.5, 500 mM NaCl, 1 mM MgCl_2_). The purities and concentrations of the TALEN proteins were measured by SDS‐PAGE (Figures 4b and S7a) and the Bradford protein assay, respectively.

### Protoplast transfection

Winter wheat variety Kenong199 was used. Wheat protoplasts were isolated from 2‐week‐old seedlings grown in nutrient‐rich soil at 25°C with a photoperiod of 16 h light: 8 h dark. The purified TALEN monomers (20 μg for each) were delivered into the protoplasts by PEG‐mediated transfection. Two days post‐transfection, the protoplasts were harvested to extract DNA for further analysis.

### Biolistic delivery of TALEN proteins

The TALEN proteins were delivered into immature wheat embryos by particle bombardment as previously described with some modifications (Liang *et al*., 2017). For each shot, the TALEN monomers (2 μg each) were premixed in Reaction Buffer (20 mM HEPES, pH 7.5, 150 mM KCl, 10 mM MgCl_2_, 0.5 mM DTT) in a total volume of 10 μL, and 5 μL of gold nanoparticles (0.6 μm) was added. The mixtures were spread directly onto the carrier and allowed to air‐dry at room temperature for about 2 h. Biolistic bombardment was performed as previously described.

## Author contributions

Z.L. and C.G. conceived the project; Z.L. and C.G. designed experiments; Z.L. performed most of the experiments; Z.L., K.C., Y.Y., Y.Z. and C.G. analysed experiments; Z.L. wrote the manuscript with assistance from other authors; and C.G. supervised the project.

## Supporting information


**Figure S1** Purification of the Cas9 variants and Cpf1 protein used in these studies.
**Figure S2** Optimization of conditions for *in vitro* cleavage by CRISPR/Cas9.
**Figure S3** Use of RNA‐guided endonucleases for indel detection.
**Figure S4** Genotyping of *tagw2* mutants induced by CRISPR/Cas9 IVTs.
**Figure S5** Genotyping of protoplast mutations induced by CRISPR/Cas9 ribonucleoprotein complexes using the PCR/RNP method.
**Figure S6** Partial sequence alignment of the three homoeologues of *TaCer9* used for mutation screening.
**Figure S7** *In vitro* cleavage of the three homoeologues of *TaGW2* using purified TALEN protein.
**Figure S8** PCR/RNP analysis of *tagw2* mutants induced by purified TALEN protein in the T0 generation.
**Figure S9** Applications of the PCR/RNP method for SNP detection.
**Figure S10** Single nucleotide mismatch cleavage assays at the OsPDS‐1 target site using the six high‐fidelity SpCas9 variants.
**Figure S11** Single nucleotide mismatch cleavage assays at the OsPDS‐4 target site using the six high‐fidelity SpCas9 variants.
**Table S1** sgRNA and crRNA target sites used for the PCR/RNP method.
**Table S2** PCR primers used in this study.
**Table S3** TALEN target loci and sequences.
