# Supplementary material for: Genotyping genome‐edited mutations in plants using CRISPR ribonucleoprotein complexes
Source: Plant Biotechnol J. 2018 May 29;16(12):2053–62. doi: 10.1111/pbi.12938 (PMC6230946; doi:10.1111/pbi.12938)
Supplement: Supplementary file 1 — Figure S1 Purification of the Cas9 variants and Cpf1 protein used in these studies. Figure S2 Optimization of conditions for in vitro cleavage by CRISPR/Cas9. Figure S3 Use of RNA‐guided endonucleases for indel detection. Figure S4 Genotyping of tagw2 mutants induced by CRISPR/Cas9 IVTs. Figure S5 Genotyping of protoplast mutations induced by CRISPR/Cas9 ribonucleoprotein complexes using the PCR/RNP method. Figure S6 Partial sequence alignment of the three homoeologues of TaCer9 used for mutation screening. Figure S7 In vitro cleavage of the three homoeologues of TaGW2 using purified TALEN protein. Figure S8 PCR/RNP analysis of tagw2 mutants induced by purified TALEN protein in the T0 generation. Figure S9 Applications of the PCR/RNP method for SNP detection. Figure S10 Single nucleotide mismatch cleavage assays at the OsPDS‐1 target site using the six high‐fidelity SpCas9 variants. Figure S11 Single nucleotide mismatch cleavage assays at the OsPDS‐4 target site using the six high‐fidelity SpCas9 variants. Table S1 sgRNA and crRNA target sites used for the PCR/RNP method. Table S2 PCR primers used in this study. Table S3 TALEN target loci and sequences. [file PBI-16-2053-s001.docx]

**Supporting Information**

**Figure S1.** Purification of the Cas9 variants and Cpf1 protein used in these studies.

**Figure S2.** Optimization of conditions for *in vitro* cleavage by CRISPR/Cas9.

**Figure S3.** Use of RNA-guided endonucleases for indel detection.

**Figure S4.** Genotyping of *tagw2* mutants induced by CRISPR/Cas9 IVTs.

**Figure S5.** *tacer9* mutations types in protoplasts detected by PCR/RE.

**Figure S6.** Partial sequence alignment of the three homoeologues of *TaCer9* used for mutation screening.

**Figure S7.** *In vitro* cleavage of the three homoeologues of *TaGW2* using purified TALEN protein.

**Figure S8.** PCR/RNP analysis of *tagw2* mutants induced by purified TALEN protein in the T0 generation.

**Figure S9.** Applications of the PCR/RNP method for SNP detection.

**Figure S10.** Single nucleotide mismatch cleavage assays at the OsPDS-1 target site using the six high-fidelity SpCas9 variants.

**Figure S11.** Single nucleotide mismatch cleavage assays at the OsPDS-4 target site using the six high-fidelity SpCas9 variants.

**Table S1.** sgRNA and crRNA target sites used for the PCR/RNP method.

**Table S2.** PCR primers used in this study.

**Table S3.** TALEN target loci and sequences.


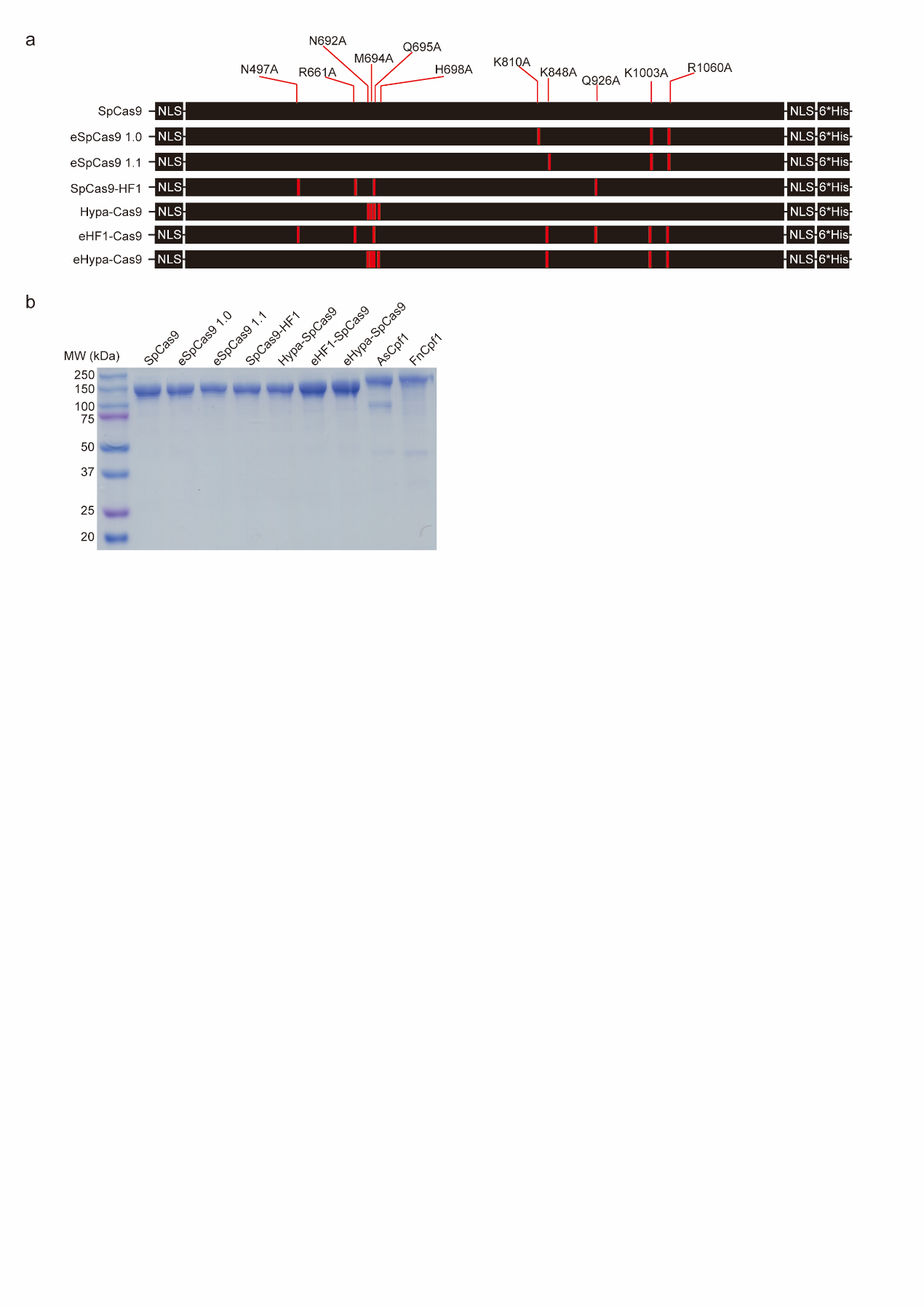


**Figure S1.** Purification of the Cas9 variants and Cpf1 protein used in these studies. (a) Schematic showing the main features of the bacterial expression plasmids harboring the various SpCas9 variants. (b) SDS-PAGE image of the purified SpCas9 protein, the high-fidelity SpCas9 variants and Cpf1 orthologs.


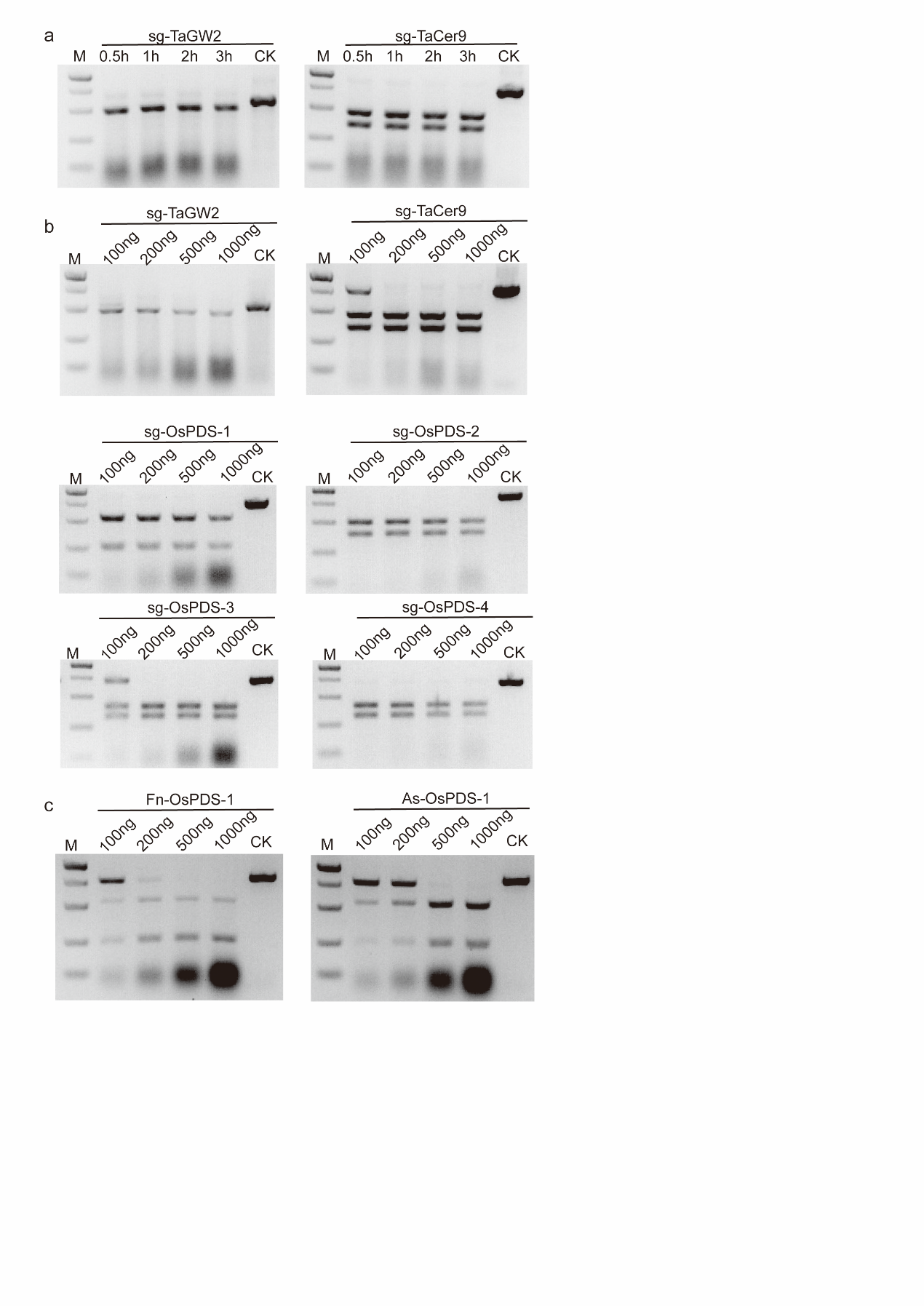


**Figure S2.** Optimization of conditions for *in vitro* cleavage by CRISPR/Cas9. (a) Optimization of the incubation time. (b) Optimization of the CRISPR/Cas9 RNP dosage used for cleavage at different target sites. (c) Optimization of the CRISPR/FnCpf1 and CRISPR/AsCpf1 RNP dosage used for cleavage at OsPDS-1 target site.


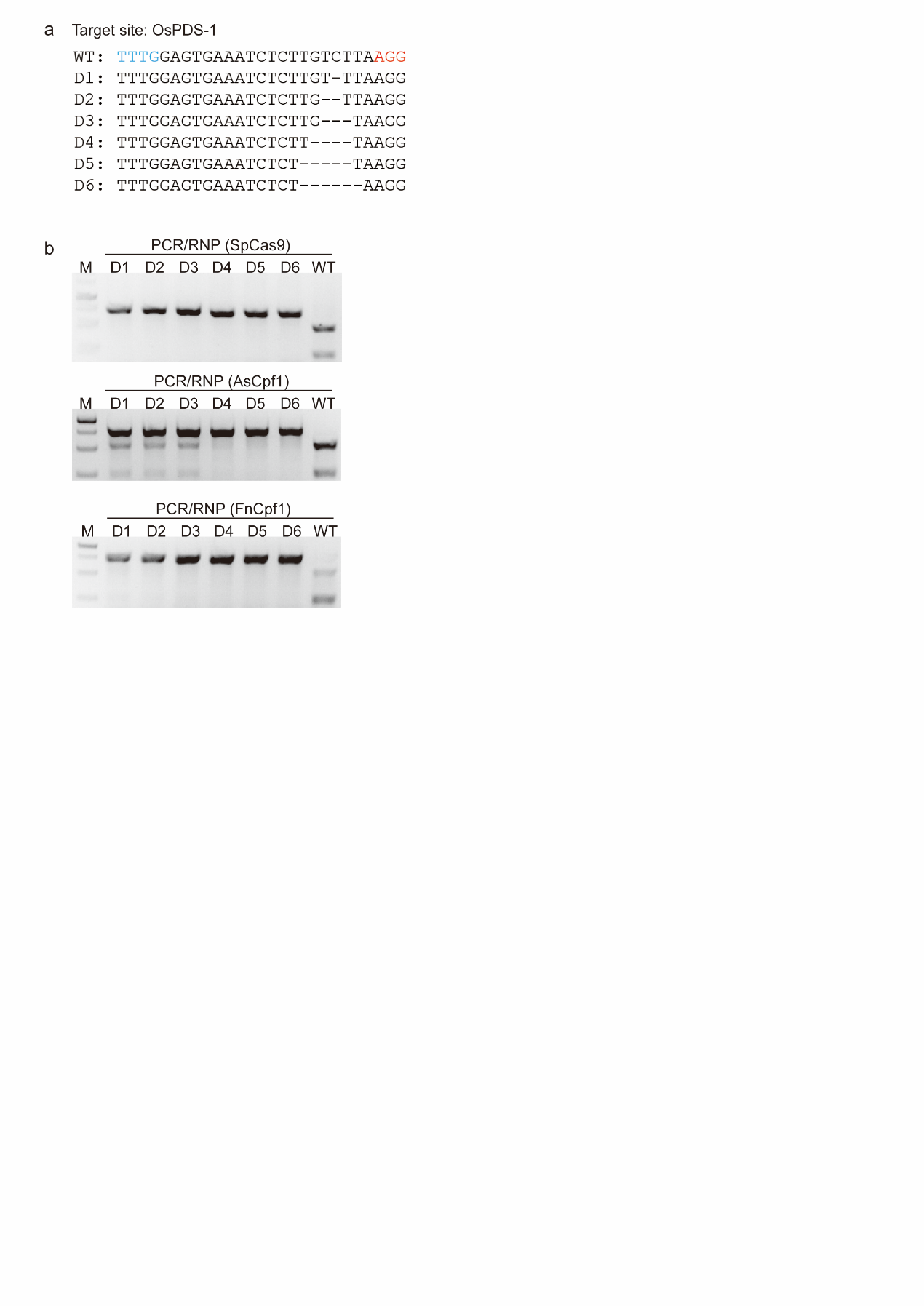


**Figure S3.** Use of RNA-guided endonucleases for indel detection. (a) The target site in exon1 of *OsPDS* . The PAM sequences of SpCas9 (5’-AGG-3’), FnCpf1 (5’-TTG-3’) and AsCpf1 (5’-TTTG-3’) are highlighted in red and blue. D1-D6 are PCR amplicons containing deletions in the target site. (b) Agarose gel analysis of *in vitro* cleavage of PCR amplicons containing target sites bearing indels with different RNA guided endonucleases.


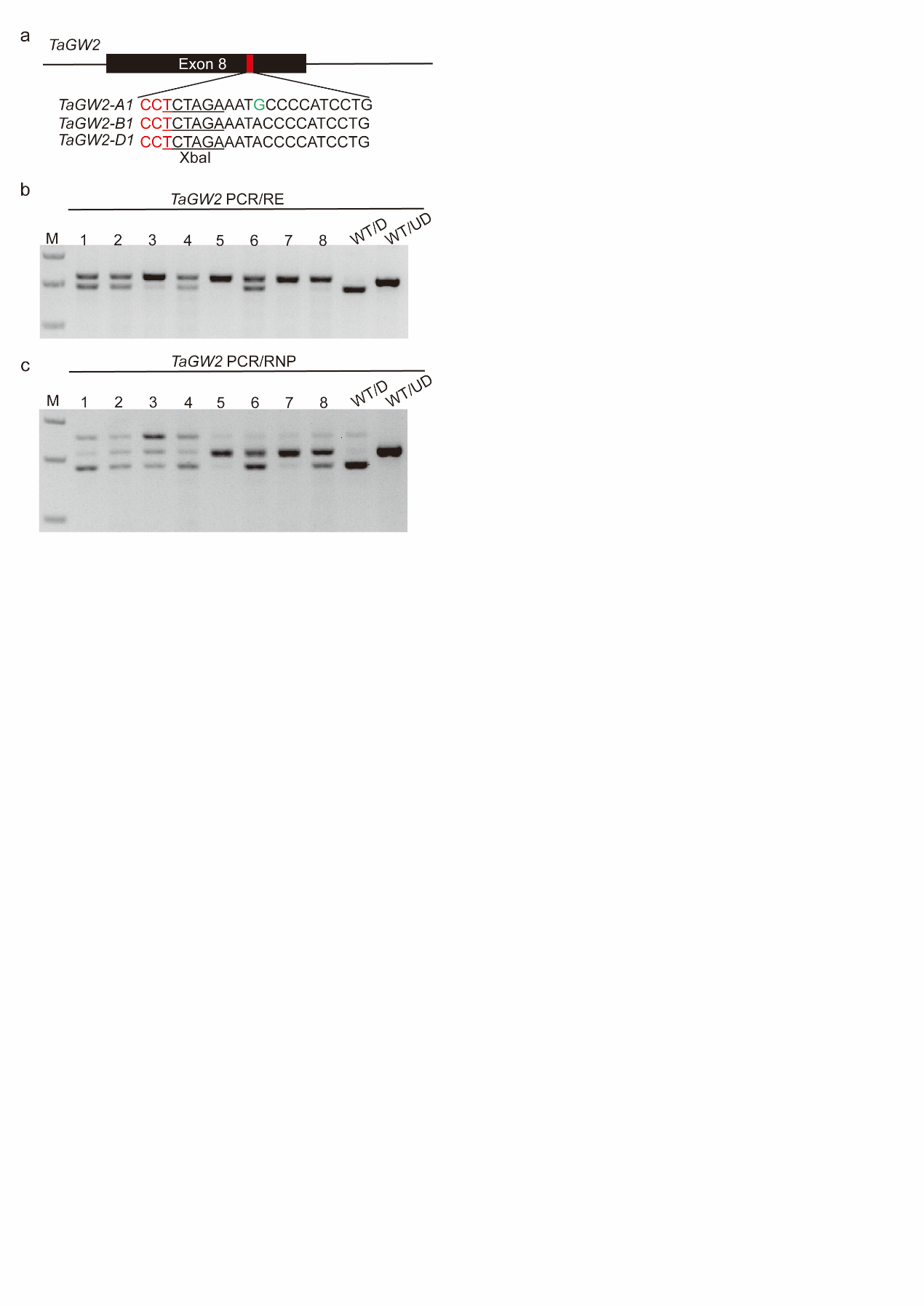


**Figure S4.** Genotyping of *TaGW2* mutants induced by CRISPR/Cas9 IVTs. (a) Sequence of the target of sg-TaGW2 in exon8 of *TaGW2.* sg-TaGW2 was used to target the three homoeologues simultaneously. The PAM sequence is highlighted in red and the XbaI restriction site is underlined. The SNP located in *TaGW2-A1* is shown in green. Agarose gel of *tagw2* mutants detected by PCR/RE (b) and PCR/RNP (c).


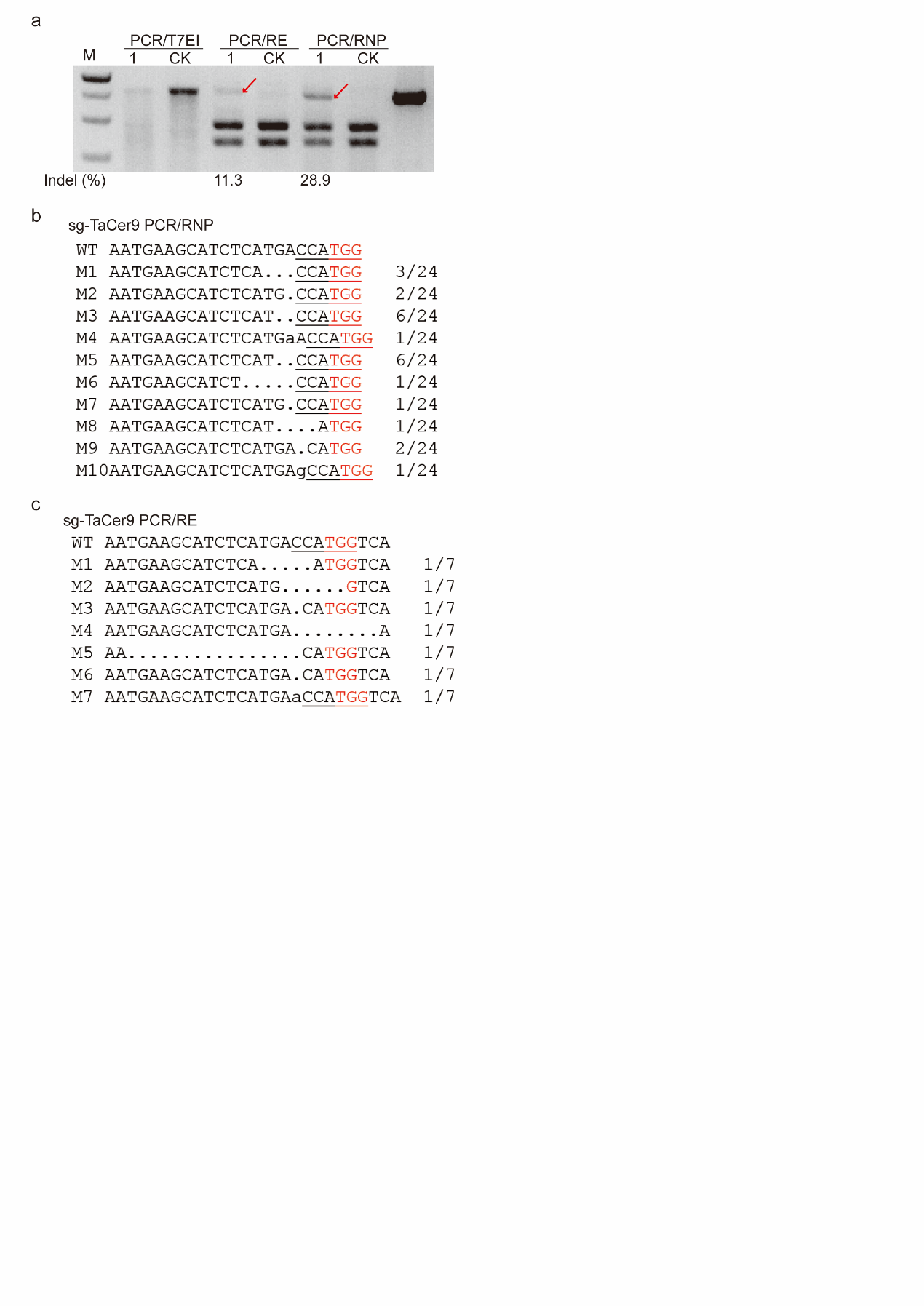


**Figure S5.** Genotyping of protoplast mutations induced by CRISPR/Cas9 ribonucleoprotein complexes using the PCR/RNP method. (a) Three different methods were used to detect mutations induced by tacer9-RNP in wheat protoplasts using conserved primer sets. Sequences of bands not cut by PCR/RNP (b) and PCR/RE (c). The PAM sequence is highlighted in red and the restriction enzyme site is underlined. The numbers on the right represent the occurrence frequency of the mutation types.


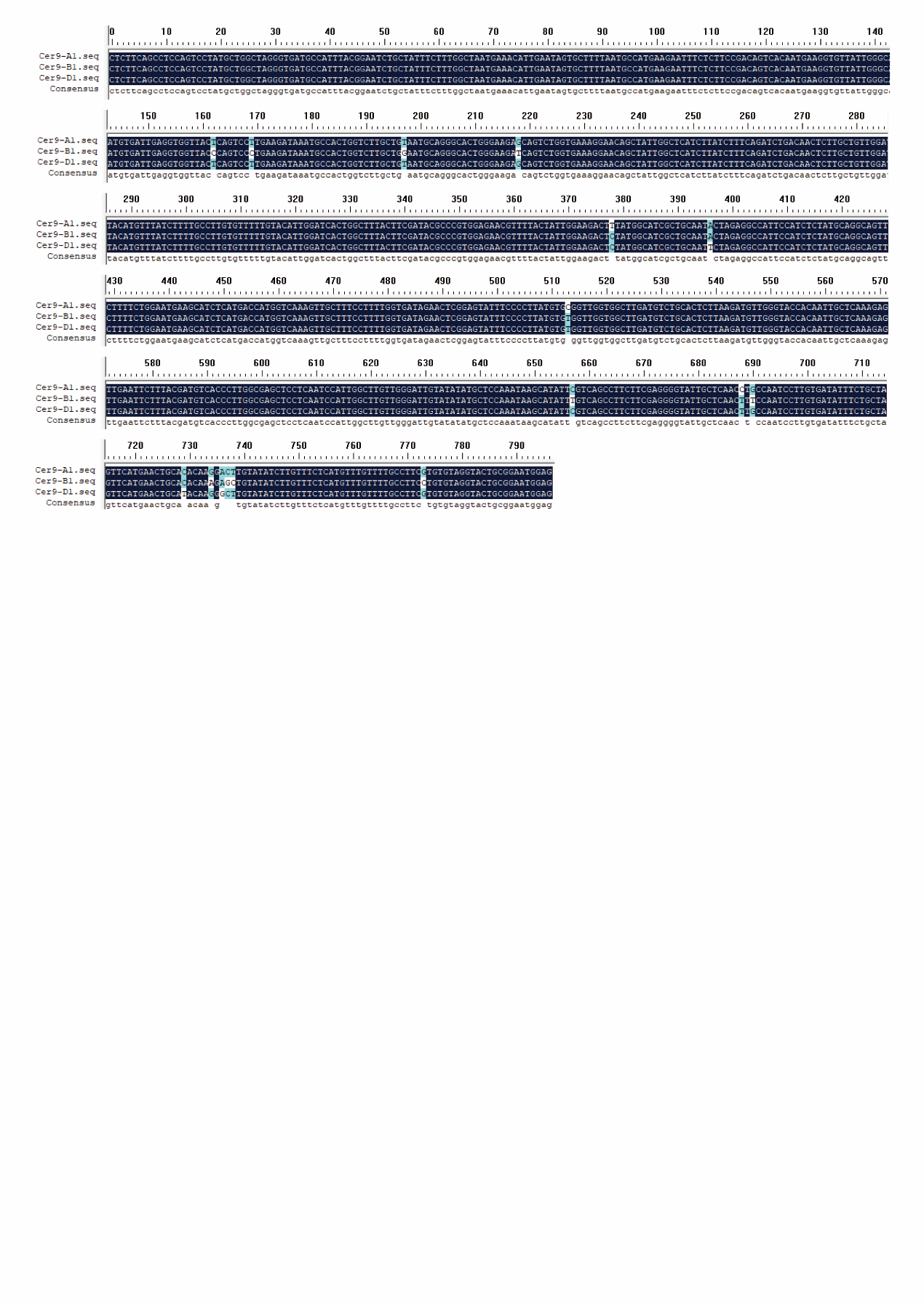


**Figure S6.** Partial sequence alignment of the three homoeologues of *TaCer9* used for mutation screening.


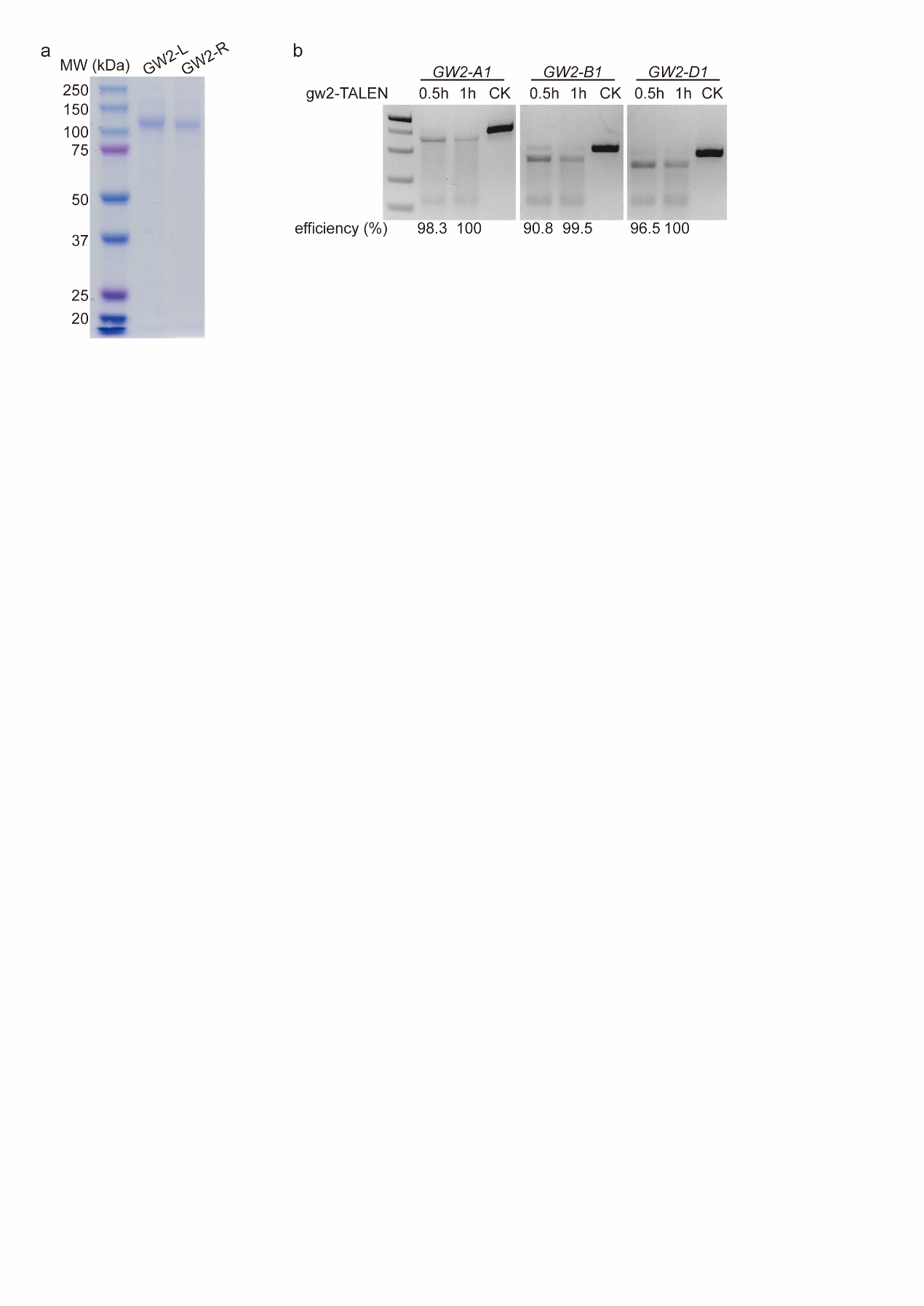


**Figure S7.** *In vitro* cleavage of the three homoeologues of *TaGW2* using purified TALEN protein. (a) SDS-PAGE gels of the purified gw2-TALEN monomers. (b) *In vitro* cleavage of *TaGW2-A1*, *-B1* and *-D1* using the gw2-TALEN protein.


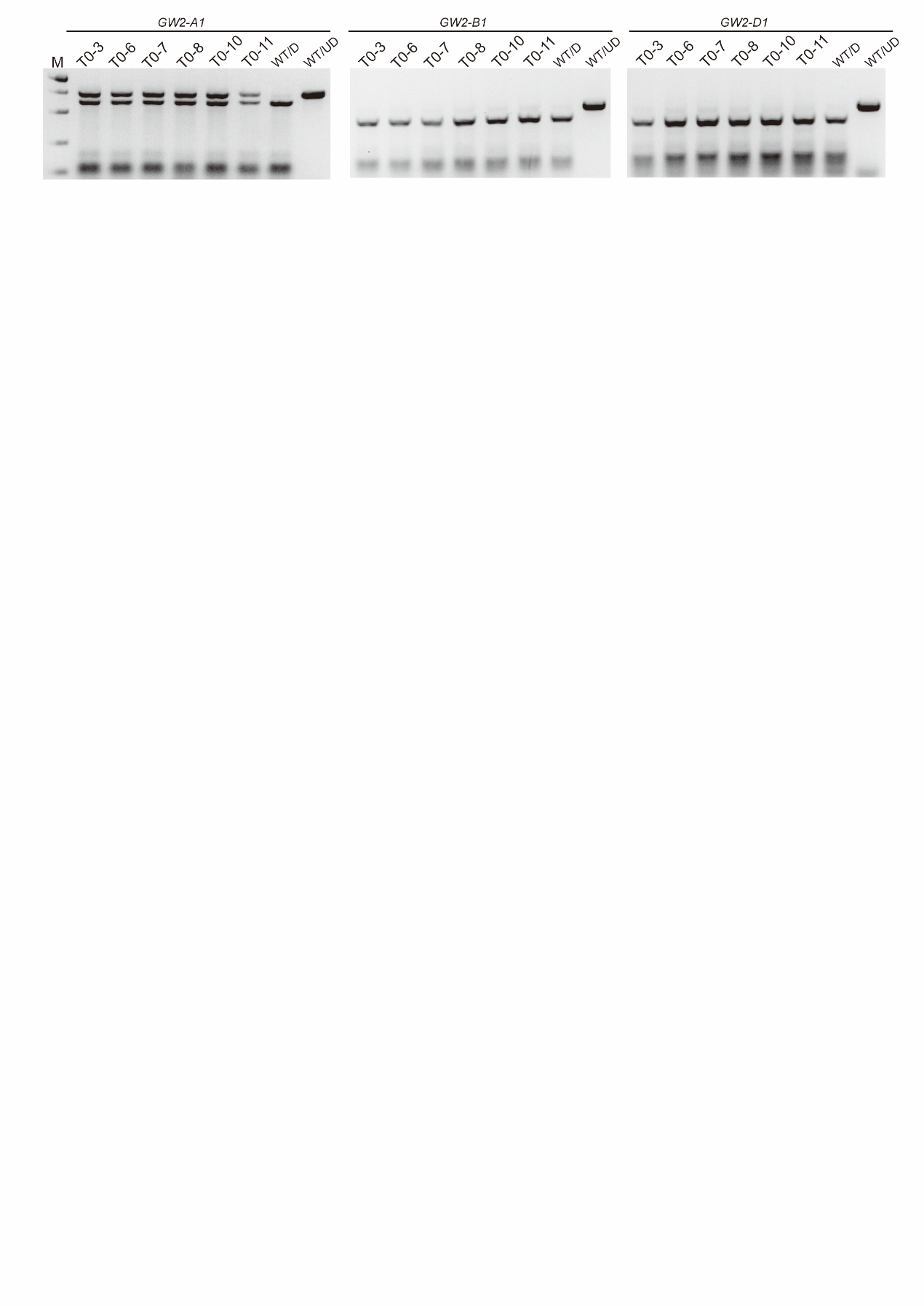


**Figure S8.** PCR/RNP analysis of *tagw2* mutants induced by purified TALEN protein in the T0 generation.


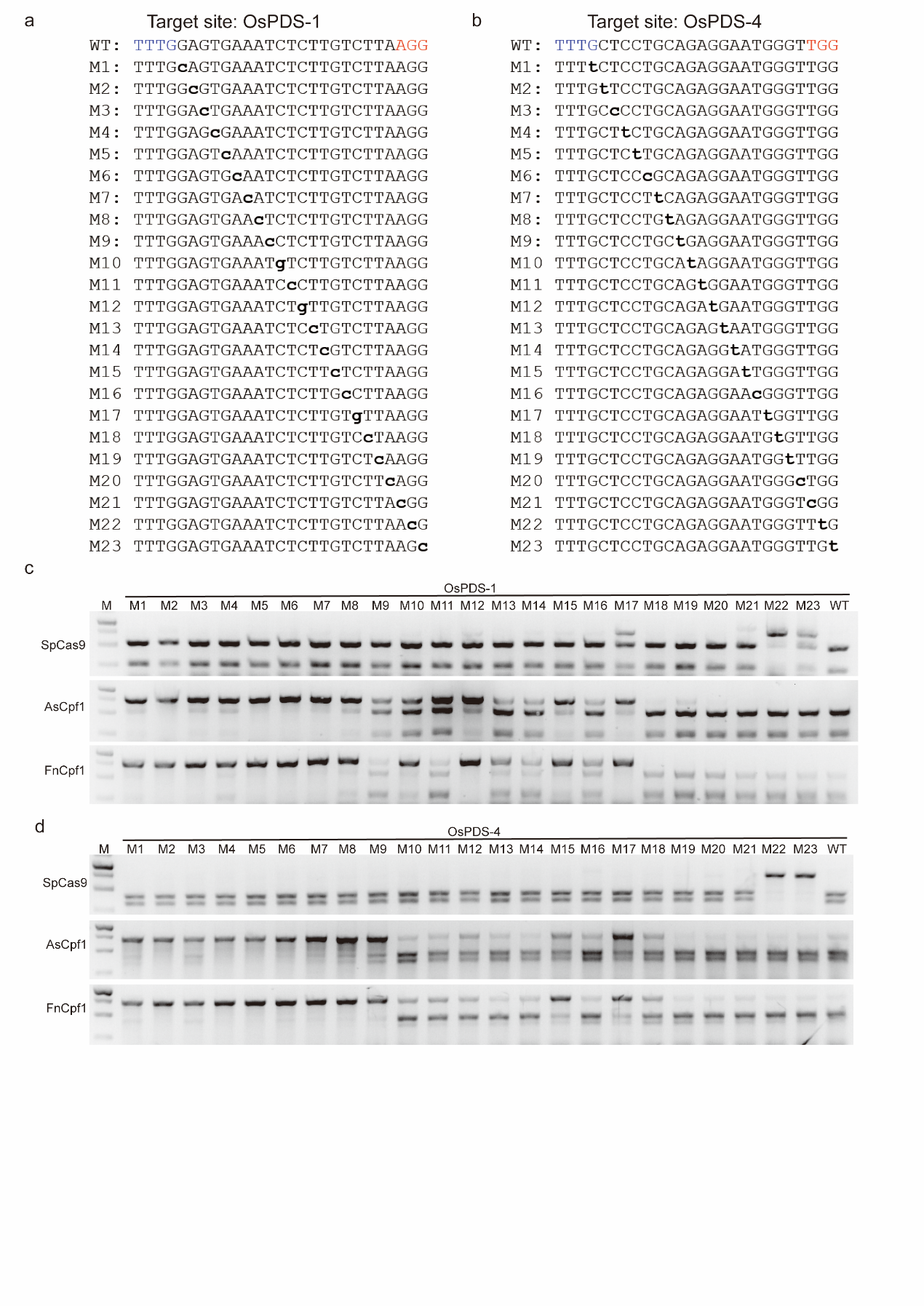


**Figure S9.** Applications of the PCR/RNP method for SNPs detection. (a and b) Single nucleotide mismatches in the OsPDS-1 and OsPDS-4 target sites. (c and d) Single nucleotide mismatch cleavage assays at the OsPDS-1 and OsPDS-4 target sites using wild-type SpCas9 variants and Cpf1 orthologues.

**
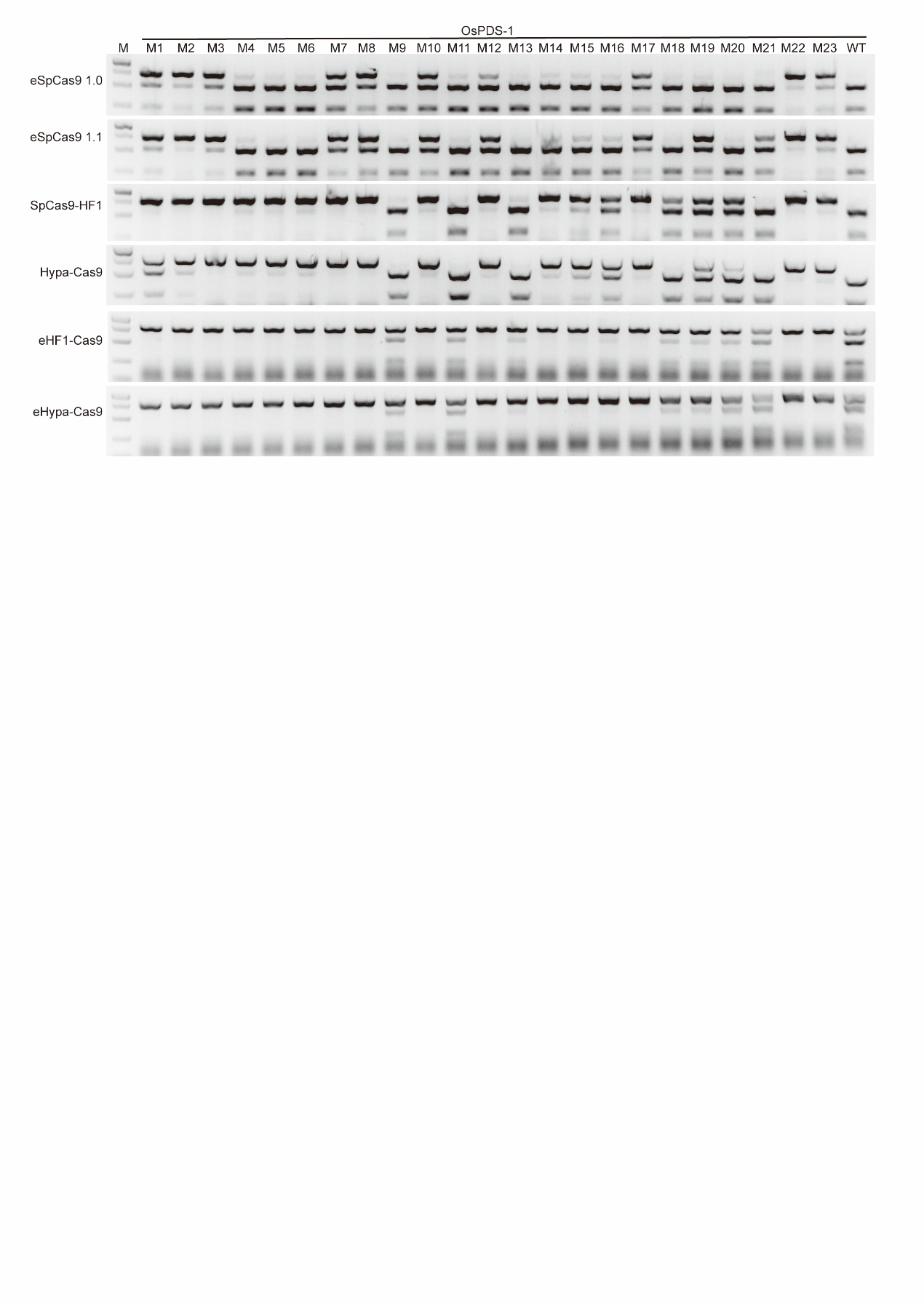
**

**Figure S10.** Single nucleotide mismatch cleavage assays at the OsPDS-1 target site using the six high-fidelity SpCas9 variants.

**
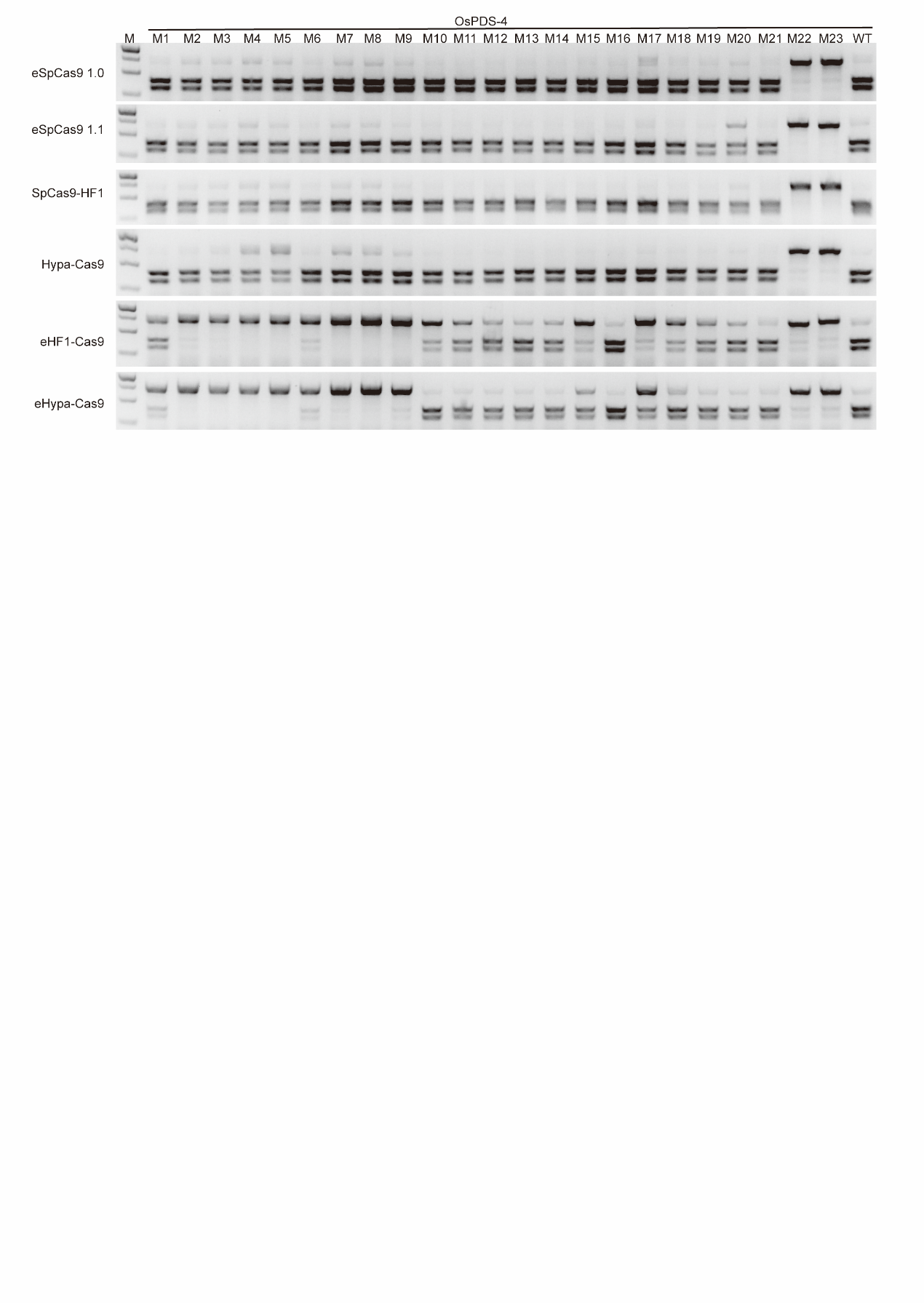
**

**Figure S11.** Single nucleotide mismatch cleavage assays at the OsPDS-4 target site using the six high-fidelity SpCas9 variants.

**Table S1.** sgRNA and crRNA target sites used for the PCR/RNP method.

| Gene Name | SSN ID | Target sequence 5’-3’ | Experiment |
| --- | --- | --- | --- |
| *TaGW2* | sg-TaGW2 | CAGGATGGGGTATTTCTAGAGG | Figure S2, S4 |
| *TaCer9* | sg-TaCer9 | AATGAAGCATCTCATGACCATGG | Figure 2, 3, S2, S4 |
| *OsCer9* | sg-OsCer9 | AACAGGGTTCCTGCTGCAGATGG | Figure 2 |
| *TaGASR7* | sg-TaGASR7 | GTTGCCGTAGGTGCCCGGCGG | Figure 2 |
| *TaGASR7* | Fn-TaGASR7 | TTGCCGTAGGTGCCCGGCGGCACGCA | Figure 2 |
| *TaGW2* | sg-TaGW2-TALEN | CCAGTCTTTGACATGTTCCGCCG | Figure 4c, 4e, and S8 |
| *TaGW2* | Fn-TaGW2-TALEN | TTTGACATGTTCCGCCGACCGTGCAA | Figure 4c |
| *TaGW2* | As-TaGW2-TALEN | TTTGACATGTTCCGCCGACCGTGCAAC | Figure 4c |
| *OsPDS* | sg-OsPDS-1 | GAGTGAAATCTCTTGTCTTAAGG | Figure 1, 5, S2, S3, S9, S10 |
| *OsPDS* | Fn-OsPDS-1 | TTGGAGTGAAATCTCTTGTCTTAAGG | Figure 5, S3, S9 |
| *OsPDS* | As-OsPDS-1 | TTTGGAGTGAAATCTCTTGTCTTAAGG | Figure S3, S9 |
| *OsPDS* | sg-OsPDS-2 | TAATGATCGGTTGCAATGGAAGG | Figure S2 |
| *OsPDS* | sg-OsPDS-3 | CTTTGCTCCTGCAGAGGAATGGG | Figure S2 |
| *OsPDS* | sg-OsPDS-4 | GCTCCTGCAGAGGAATGGGTTGG | Figure 5, S2, S9, S11 |
| *OsPDS* | Fn-OsPDS-4 | TTGCTCCTGCAGAGGAATGGGTTGG | Figure 5, S9 |
| *OsPDS* | As-OsPDS-4 | TTTGCTCCTGCAGAGGAATGGGTTGG | Figure S9 |

The PAM sequence of each target site is underlined.

**Table S2.** PCR primers used in this study.

| Primer name | Primer sequence | Application |
| --- | --- | --- |
| GW2-F  GW2-R | ATGCCAACCCTTGCGTGTGCGT  TCCTGCTTGTGGGAGCTTTATG | Amplifying the *TaGW2* target site of gw2-TALEN and sg-TaGW2 |
| GW2-A1-F | CTGCCATTACTTTGTATTTTGGTAATA | Forward primer to amplify the *TaGW2-A1* target site of gw2-TALEN and sg-TaGW2 |
| GW2-B1-F | GTTCAGATGGCAATCTAAAAGTT | Forward primer to amplify the *TaGW2-B1* target site of gw2-TALEN and sg-TaGW2 |
| GW2-D1-F | GCATGTACTTTGATTGTTTGCGTGA | Forward primer to amplify the *TaGW2-D1* target site of gw2-TALEN and sg-TaGW2 |
| GW2-A1/B1/D1-R | TCCTTCCTCTCTTACCACTTCCC | Amplifying the *TaGW2-A1, -B1 and -D1* target site of gw2-TALEN and sg-TaGW2 |
| OsPDS-1F  OsPDS-1R | GCAGCCACATAGAGAAACTCG  GCAATCACGACCTGTAATGGT | Amplifying the *OsPDS* target site of sg/Fn/As-OsPDS-1 |
| OsPDS-2F  OsPDS-2R | TAGGTTATTACCGCCAGCAC  GTCCAAGAGCAAACTTCACC | Amplifying the *OsPDS* target site of sg-OsPDS-2 |
| OsPDS-3+4F  OsPDS-3+4R | GGGTTTACCATCTTCAAGCATC  CACTACAGACTGAGCACAAAGC | Amplifying the *OsPDS* target site of sg-OsPDS-3 and sg/Fn/As-OsPDS-4 |
| TaCer9-F | CTCTTCAGCCTCCAGTCCTAT | Forward primer to amplify the *TaCer9-A1, -B1 and -D1* target site of sg-TaCer9 |
| TaCer9-R | CTCCATTCCGCAGTACCTACA | Reverse primer to amplify the *TaCer9-A1, -B1 and -D1* target site of sg-TaCer9 |
| TaCer9-A1R | GAGGGGCTTCAACAGAACAC | Reverse primer to amplify the *TaCer9-A1* target site of sg-TaCer9 |
| TaCer9-B1R | ACATGAGAAACAAGATATACAGCTCT | Reverse primer to amplify the *TaCer9 -B1* target site of sg-TaCer9 |
| TaCer9-D1R | GGGAGGGGACTTCAAGAAAAC | Reverse primer to amplify the *TaCer9 -D1* target site of sg-TaCer9 |
| GASR7-F  GASR7-R | GGAGGTGATGGGAGGTGGGGG  CTGGGAGGGCAATTCACATGCCA | Amplifying the *TaGASR7* target site of sg/Fn-TaGASR7 |
| OsCer9-F  OsCer9-R | TCGTGAGAAGTCTTGGTGAAG  TGTGGGACGGAGGAAGTAATA | Amplifying the *OsCer9* target site of sg-OsCer9 |
| MLO-F  MLO-R | GTCTTCGCCGTCATGATCATCGTCTCC  TGGTATTCCAAGGAGGCGGTCTCTGTCT | Amplifying the *TaMLO* target site of mlo-TALEN |
| MLO-A1F  MLO-A1R | TGGCGCTGGTCTTCGCCGTCATGATCATCGTC  TACGATGAGCGCCACCTTGCCCGGGAA | Amplifying the *TaMLO-A1* target site of mlo-TALEN |
| MLO-B1F  MLO-B1R | ATAAGCTCGGCCATGTAAGTTCCTTCCCGG  CCGGCCGGAATTTGTTTGTGTTTTTGTT | Amplifying the *TaMLO-B1* target site of mlo-TALEN |
| MLO-D1F  MLO-D1R | TGGCTTCCTCTGCTCCCTTGGTGCACCT  TGGAGCTGGTGCAAGCTGCCCGTGGACATT | Amplifying the *TaMLO-D1* target site of mlo-TALEN |
| T7-Sp-GW2-TALEN-F | TAATACGACTCACTATAGGGGCGGAACATGTCAAAGACgttttagagctagaaatagc | Forward primer to amplify the T7-GW2-TALEN-sgRNA |
| T7-Sp-GW2-F | TAATACGACTCACTATAGGCAGGATGGGGTATTTCTAG | Forward primer to amplify the T7-GW2-sgRNA |
| T7-Sp-GASR7-F | TAATACGACTCACTATAGGGTTGCCGTAGGTGCCCGG | Forward primer to amplify the T7-GASR7-sgRNA |
| T7-Sp-TaCer9-F | TAATACGACTCACTATAGGATGAAGCATCTCATGACCA | Forward primer to amplify the T7-TaCer9-sgRNA |
| T7-Sp-OsCer9-F | TAATACGACTCACTATAGGACAGGGTTCCTGCTGCAGA | Forward primer to amplify the T7-OsCer9-sgRNA |
| T7-Sp-PDS-1F | TAATACGACTCACTATAGGAGTGAAATCTCTTGTCTTA | Forward primer to amplify the T7-OsPDS-1-sgRNA |
| T7-Sp-PDS-2F | TAATACGACTCACTATAGGAATGATCGGTTGCAATGGA | Forward primer to amplify the T7-OsPDS-2-sgRNA |
| T7-Sp-PDS-3F | TAATACGACTCACTATAGGTTTGCTCCTGCAGAGGAAT | Forward primer to amplify the T7-OsPDS-3-sgRNA |
| T7-Sp-PDS-4F | TAATACGACTCACTATAGGCTCCTGCAGAGGAATGGGT | Forward primer to amplify the T7-OsPDS-4-sgRNA |
| sgRNA-PCR-R | GCACCGACTCGGTGCCACTT | Reverse primer to amplify the T7-XX-sgRNA |
| T7-As-F | TAATACGACTCACTATAGGGTAATTTCTACTCTTGTAGAT | Forard primer to amplify the T7-XX-As-crRNA |
| As-GW2-TALEN-R | GTTGCACGGTCGGCGGAACATGTATCTACAAGAGTAGAAATTA | Reverse primer to amplify the T7-GW2-TALEN-As-crRNA |
| As-PDS-1R | CCTTAAGACAAGAGATTTCACTCATCTACAAGAGTAGAAATTA | Reverse primer to amplify the T7-OsPDS-1-As-crRNA |
| As-PDS-4R | TCCAACCCATTCCTCTGCAGGAGATCTACAAGAGTAGAAATTA | Reverse primer to amplify the T7-OsPDS-4-As-crRNA |
| T7-Fn-F | TAATACGACTCACTATAGGGTAATTTCTACTGTTGTAGAT | Forard primer to amplify the T7-XX-Fn-crRNA |
| Fn-GW2-TALEN-R | TTTGACATGTTCCGCCGACCGTGATCTACAACAGTAGAAATTA | Reverse primer to amplify the T7-GW2-TALEN-Fn-crRNA |
| Fn-GASR7-R | TGCGTGCCGCCGGGCACCTACGGATCTACAACAGTAGAAATTA | Reverse primer to amplify the T7-GASR7-Fn-crRNA |
| Fn-PDS-1R | CCTTAAGACAAGAGATTTCACTCATCTACAACAGTAGAAATTA | Reverse primer to amplify the T7-OsPDS-1-Fn-crRNA |
| Fn-PDS-4R | TCCAACCCATTCCTCTGCAGGAGATCTACAACAGTAGAAATTA | Reverse primer to amplify the T7-OsPDS-4-Fn-crRNA |

**Table S3.** TALEN target loci and sequences.

| Gene Name | Target sequence | Detection method |
| --- | --- | --- |
| *TaGW2* | TAGTAGTGCGTTCCCAGTCtttgacatgttccgccgaCCGTGCAACATTGCTGGTGGA | PCR/RNP (SpCas9) |
